# Graft dysfunction in chronic antibody-mediated rejection correlates with B-cell–dependent indirect antidonor alloresponses and autocrine regulation of interferon-γ production by Th1 cells

**DOI:** 10.1016/j.kint.2016.10.009

**Published:** 2017-02

**Authors:** Kin Yee Shiu, Laura McLaughlin, Irene Rebollo-Mesa, Jingyue Zhao, Hannah Burton, Harriet Douthwaite, Hannah Wilkinson, Vikki Semik, Philippa C. Dodd, Paul Brookes, Robert I. Lechler, Maria P. Hernandez-Fuentes, Claudia Kemper, Anthony Dorling

**Affiliations:** 1 MRC Centre for Transplantation, King’s College London, Guy’s Hospital, London, UK; 2 Imperial College London and Imperial College NHS Trust, Hammersmith Hospital, London, UK

**Keywords:** B lymphocyte, chronic allograft nephropathy, ELISPOT, indirect alloresponses, interferon-γ

## Abstract

Chronic antibody-mediated rejection, a common cause of renal transplant failure, has a variable clinical phenotype. Understanding why some with chronic antibody-mediated rejection progress slowly may help develop more effective therapies. B lymphocytes act as antigen-presenting cells for *in vitro* indirect antidonor interferon-γ production in chronic antibody-mediated rejection, but many patients retain the ability to regulate these responses. Here we test whether particular patterns of T and B cell antidonor response associate with the variability of graft dysfunction in chronic antibody-mediated rejection. Our results confirm that dynamic changes in indirect antidonor CD4^+^ T-cell responses correlate with changes in estimated glomerular filtration rates, independent of other factors. Graft dysfunction progressed rapidly in patients who developed unregulated B-cell–driven interferon-γ production. However, conversion to a regulated or nonreactive pattern, which could be achieved by optimization of immunosuppression, associated with stabilization of graft function. Functional regulation by B cells appeared to activate an interleukin-10 autocrine pathway in CD4^+^ T cells that, in turn, impacted on antigen-specific responses. Thus, our data significantly enhance the understanding of graft dysfunction associated with chronic antibody-mediated rejection and provide the foundation for strategies to prolong renal allograft survival, based on regulation of interferon-γ production.

Kidney transplantation is the best treatment for kidney failure, in terms of length and quality of life and cost-effectiveness,^1^, ^2^ but a significant number of patients keep their transplants for less than 10 years,^3^ returning to dialysis as the transplant fails. The single biggest cause is immune-mediated injury.^4^ The association between antibody (Ab) against donor human leukocyte antigen (HLA) (donor-specific Ab [DSA]) and graft failure,^5^ and description of specific histological features constituting antibody-mediated rejection (AMR),^6^ have advanced our understanding of this problem. Graft failure is usually preceded by a progressive decline in glomerular filtration rate (GFR), although many patients with DSA have stable graft function, and the immunological factors that influence decline in GFR are unknown.

We recently reported the findings of a long-term observational study in patients with a transplant biopsy diagnosis of chronic AMR (CAMR),^7^ describing the activity of antidonor T cells recognizing donor antigen via the indirect pathway.^8^ For the first time, we showed that donor antigen presentation by B cells in enzyme-linked immunosorbent spot (ELISPOT) assays of interferon (IFN)-γ production by CD8-depleted peripheral blood mononuclear cells (PBMCs) was seen preferentially in patients with CAMR compared with controls. Importantly, two-thirds of nonreactive samples had evidence of suppression of antidonor IFN-γ production by CD19^+^ B lymphocytes or CD25^+^ T cells, challenging the prevalent hypothesis that patients with chronic rejection have lost the ability to regulate antidonor cellular immunity.^9^ In this report, we expand our findings from the same cohort by describing the dynamic changes in ELISPOT patterns in individual patients and report an association with changes in estimated GFR (eGFR), testing the hypothesis that progression of renal dysfunction is influenced by the activity of antidonor cell-mediated responses. We provide evidence of the predictive accuracy of ELISPOT, above that provided by other clinical factors alone. Finally, in attempting to demonstrate the role of interleukin (IL)-10 in patients with regulated ELISPOT responses, we discovered evidence that B cells activated a well-defined IL-10 autocrine regulatory mechanism in T helper 1 (Th-1) cells, which was involved in suppressing antidonor responses. Further investigation of the importance of cellular immune responses in AMR may promote a deeper understanding of how to treat chronic rejection.

## Results

### Patient groups and outcomes

**Figure 1:**
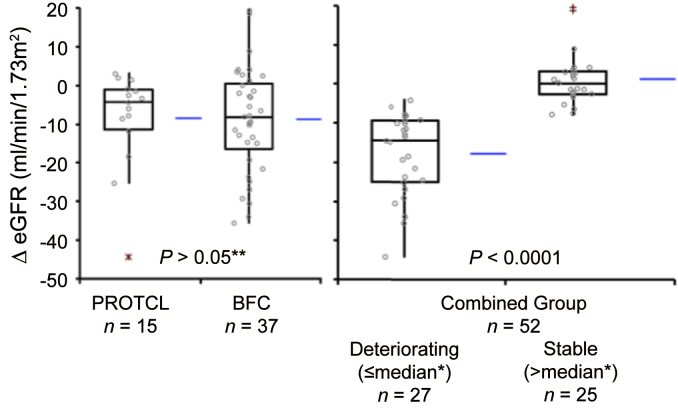
**ΔeGFR in PROTCL and BFC subgroups and combined group.** Box plots show median and IQR, with whiskers representing data within 1.5 the IQR of the upper and lower quartiles, with outliers >1.5 and <3.0 IQR as + and >3.0 IQR as *. Horizontal lines to right of box plots indicate the mean value. Nine patients with BFC who either had missing follow-up data (*n* = 2), or eGFR <20 ml/min per 1.73 m^2^ at time of biopsy (*n* = 7) have been excluded (Supplementary Table S1c). The “combined” group includes all patients with PROTCL and BFC, and have been split into “deteriorating” and “stable” subgroups, based on the relationship to the median ΔeGFR in each of the PROTCL and BFC subgroups. *Deteriorating group contains patients with ΔeGFR below or equal to the median in each of PROTCL and BFC groups (*n* = 27). The median ΔeGFR in this subgroup is −14.4 ml/min per 1.73 m^2^ (IQR 15.5). Stable group contains patients with ΔeGFR above the median from each of the PROTCL and BFC groups (*n* = 25). The median ΔeGFR for this group is 0.3 ml/min per 1.73 m^2^ (IQR 6.0). **Mann-Whitney *U* test.

This report concerns 52 patients included in our recent publication^7^ who had either a protocol (PROTCL, *n* = 15) or “for-cause” biopsy (BFC, *n* = 37). Reasons for exclusions and relevant details of those included are provided in the Supplementary Material. Blood samples were collected within a month of biopsy (time point 1) and 9 to 12 months later (time point 2) for analysis of DSA and antidonor IFN-γ responses. There were no graft failures in the PROTCL group, whereas 11 grafts from the BFC group failed. There was no statistically significant difference in median eGFR at time of first ELISPOT between the 11 who had graft failure (39.3 ml/min per 1.73 m^2^ [interquartile range (IQR) 16.8]) and the other 41 who maintained graft function during the course of the study (45.7 ml/min per 1.73 m^2^ [IQR 23.9] *P* = 0.1 Mann-Whitney *U* test). To assist interpretation of some analyses, changes in eGFR (ΔeGFR) were dichotomized into “deteriorating” (*n* = 27) and “stable” (*n* = 25), based on relationship to the median in each of the PROTCL or BFC groups (Figure 1, Supplementary Tables S1B and S1C), and the 2 subgroups created had statistically significant differences in ΔeGFR, despite having similar eGFRs at the time of first ELISPOT (Supplementary Tables S1B and S1C). All 11 patients who lost graft function were in the “deteriorating” subgroup.

### Associations with *ΔeGFR*

#### Proteinuria, biopsy features, and DSA (Table 1)

Proteinuria at the time of biopsy was strongly associated with graft failure, and a protein-to-creatinine ratio >50 was a sensitive marker of graft failure, whereas protein-to-creatinine ratio <50 was highly predictive of graft survival (see legend to Table 1). Protein-to-creatinine ratio was also associated with ΔeGFR, although was relatively insensitive and poorly predictive of whether a patient was “stable” or “deteriorating.” Two specific biopsy features were associated with graft failure, but both appeared relatively insensitive and poorly predictive within the follow-up period. There was no association between ΔeGFR and either of these biopsy features (Supplementary Figure S1 and Supplementary Table S2). The mean fluorescence intensities of serum DSA and lack of association with ELISPOT activity were presented in Shiu *et al.*^7^ DSAs were associated with graft failure, but with poor sensitivity, and although they were also associated with ΔeGFR at time point 1 (Supplementary Figure S2), they could not discriminate between stable and deteriorating subgroups, all suggesting that DSA presence was not sensitive, predictive, or relatively specific at discriminating patients with outcomes based on ΔeGFR.

#### ELISPOT patterns (Table 2, Table 3, Table 4, Table 5)

**Figure 2:**
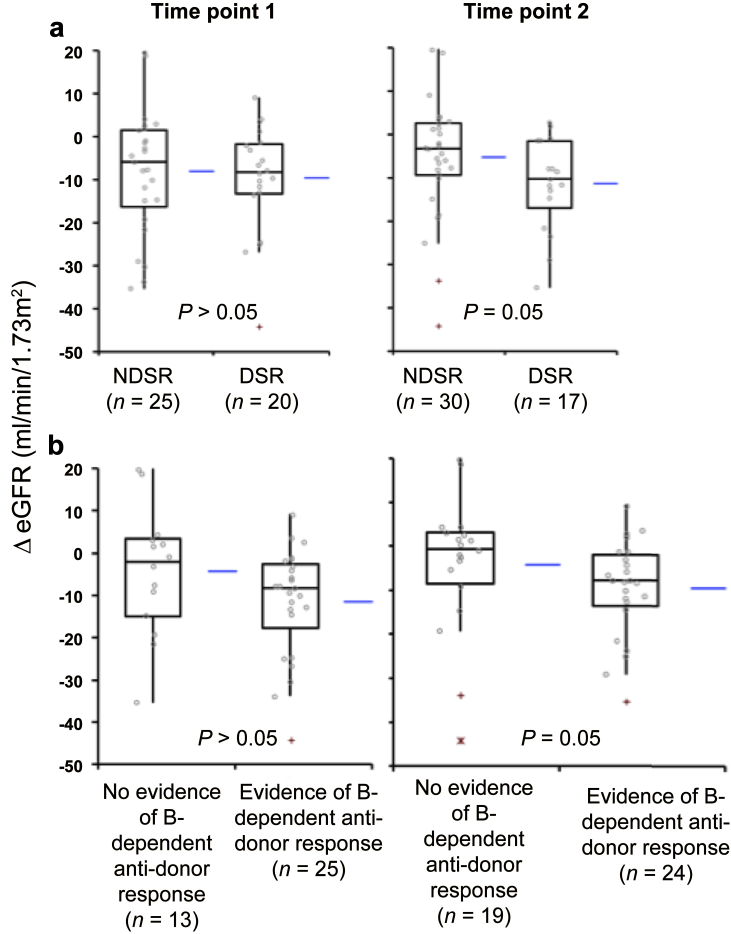
**Association between ELISPOT patterns and ΔeGFR over the course of the study in combined PROTCL and BFC group.** Box plots show median and IQR, with whiskers representing data within 1.5 of the IQR of the upper and lower quartiles, with outliers >1.5 and <3.0 IQR as + and >3.0 IQR as *. Horizontal lines to right of box plots indicate the mean value. (**a**) Patterns grouped according to DSR versus NDSR status. Time point 1: Patients with ELISPOT showing DSR have median ΔeGFR of −8.0 ml/min (IQR 11.6) and mean ΔeGFR of −9.6 ml/min (SD ±12.6). Patients with NDSR have median ΔeGFR of −5.8 ml/min (IQR 17.9) and mean ΔeGFR of −8.0 ml/min (SD ±14.4). *P* = 0.70 Mann-Whitney *U*. Time point 2: Patients with ELISPOT showing DSR have median ΔeGFR of −10.1 ml/min (IQR 15.5) and mean ΔeGFR of −11.3 (±SD 10.9) ml/min. Patients with NDSR have median ΔeGFR of −3.1 ml/min (IQR 11.9) and mean ΔeGFR of −5.1 ml/min (SD ±13.4). *P* = 0.05 Mann-Whitney *U*. NB: Analysis with 2 outliers at time point 2 removed (ΔeGFR −33.7 [ID 392] and 44.2 [ID 958] both in NDSR group) and replaced with missing data reveal *P* = 0.015. (**b**) Patterns group according to evidence on ELISPOT of B-cell–dependent antidonor reactivity. Time point 1: Patients with ELISPOTs showing evidence of B-dependent antidonor IFN-γ production have median ΔeGFR of −8.3 ml/min per 1.73 m^2^ (IQR 15.2) and mean ΔeGFR of −11.5 (SD ±15.0 ml/min per 1.73 m^2^). Patients with ELISPOT showing no evidence of B-dependent IFN-γ production have median ΔeGFR of −0.9 ml/min (IQR 17.9) and mean ΔeGFR of −4.0 (SD ±15.6) ml/min. *P* > 0.11 Mann-Whitney *U*. Time point 2: Patients with ELISPOTs showing evidence of B-dependent IFN-γ production have median ΔeGFR of −7.9 ml/min per 1.73m^2^ (IQR 11.7) and mean ΔeGFR of −9.6 (SD ±10.9 ml/min per 1.73 m^2^). Patients with ELISPOT showing no evidence of B-dependent IFN-γ production have median ΔeGFR of −0.9 ml/min (IQR 11.6) and mean ΔeGFR of −4.1 (SD ±15.4) ml/min. *P* = 0.053 Mann-Whitney *U*. NB: Analysis with 3 outliers at time point 2 removed (ΔeGFR −33.7 [ID 392] and −44.2 [ID 958] both in “No evidence of B-dependency” group, and ΔeGFR −35.3 [ID 635] in “Evidence of B-dependency” group) and replaced with missing data reveal *P* = 0.01.

Donor-specific reactivity (DSR) was defined as antidonor reactivity above threshold (see Tables 2 and 3) by CD8-depleted PBMCs in IFN-γ ELISPOT assays, after incubation with whole donor-derived proteins, whereas no DSR (NDSR) refers to a subthreshold response. Tables 2 and 3 show the different types of patterns obtained and the numbers of samples with each pattern at time points 1 and 2. In the whole cohort, the greatest loss of GFR was seen in patients with DSR at time point 2 (Figure 2a) and approximately 65% of patients with DSR at this time point appeared in the “deteriorating subgroup”; these relationships just failed to reach statistical significance. However, when analyzed separately, DSR at time point 2 was associated with deterioration in the BFC but not the PROTCL subgroup (Supplementary Figure S3 and Supplementary Table S3).

ELISPOT patterns also could be defined according to functional B-cell phenotype and in particular whether there was evidence of a B-cell–dependent antidonor response. Using this definition, associations between ΔeGFR and ELISPOT patterns were maintained (Figure 2b, Table 5). Subgroup analysis suggested a strengthening of associations in the PROTCL patients at time point 1, whereas in the BFC subgroup, associations at time point 2 were weakened and failed to reach statistical significance (Supplementary Figure S4 and Supplementary Table S4).

#### Multivariate logistic regression analysis

**Figure 3:**
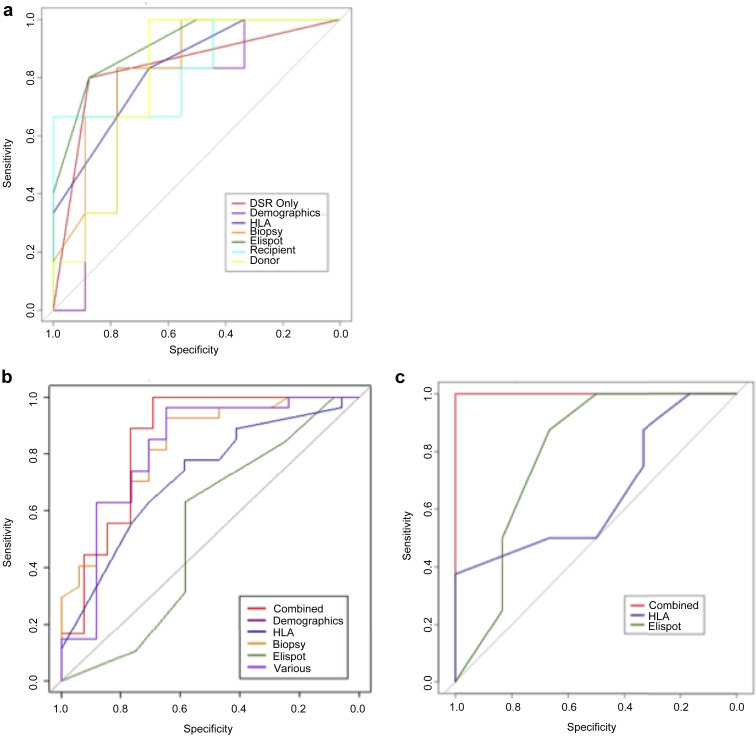
**Multivariate logistic regression models in patient subgroups.** ROC curves corresponding to the multivariate logistic regression models for linked groups of predictive variable in the PROTCL biopsy (**a**), BFC (**b**), and the optimized treatment BFC-CAMR subgroup with deteriorating creatinines (**c**), using generalized linear models to estimate each of the models, followed by elastic net estimate the optimal combined algorithm, with cross validation for parameter tuning. The predictive variables included in each of the models are listed in Supplementary Tables S5 and S6.

The independent predictive value of the ELISPOT assay at time point 1 was considered separately in PROTCL and BFC subgroups, by comparison with other factors potentially associated with graft dysfunction. We estimated a series of multivariate logistic regression models, each of which included groups of related predictive variables: demographics, recipient factors from time of transplantation, donor factors, HLA antibody, protocol biopsy features, and ELISPOT results (Supplementary Tables S5 and S6). The probability of graft dysfunction as estimated by each of the models was then used to build receiver operator characteristic curves to evaluate performance differences across the different models. In the PROTCL group, ELISPOT assay results alone were able to predict development of graft dysfunction better than any other set of risk factors (Supplementary Table S5). Subsequently, elastic net with leave-group-out cross-validation was used to select the optimal model for classification, considering all predictors in a combined model. Results showed that a predictive algorithm that included B-dependent DSR (from the ELISPOT assay) as the *only* predictor provided the best performance, with an area under the curve (AUC) of 0.84 (95% confidence interval 0.61–1, specificity 0.88, sensitivity 0.80) (Figure 3a). The cross-validated estimate of the AUC was 0.89.

A similar approach was used for the BFC subgroup (Supplementary Table S6), but the optimal model generated by elastic net and leave-group-out cross-validation identified 5 factors, including B-dependent DSR on ELISPOT assay (the others were HLA Ab status [including DSA mean fluorescence intensity at time of biopsy], C4d in peritubular capillaries (PTC), degree of interstitial fibrosis/tubular atrophy (IF/TA) on biopsy, and proteinuria). This combined model produced a receiver operating characteristic curve with an AUC of 0.85 (95% confidence interval 0.72–1) with a peak of 89% sensitivity and 77% specificity, which was better than any of the individual models (Figure 3b). The cross-validated estimate of the AUC was 0.73. These data indicate that the patterns of antidonor T-cell IFN-γ production, from around the time of biopsy, do have prognostic influence on progression of renal dysfunction, particularly in the PROTCL group.

### Dynamic changes in antidonor IFN-γ production and association with eGFR

#### Loss of responsiveness/regulation and dysfunction

**Figure 4:**
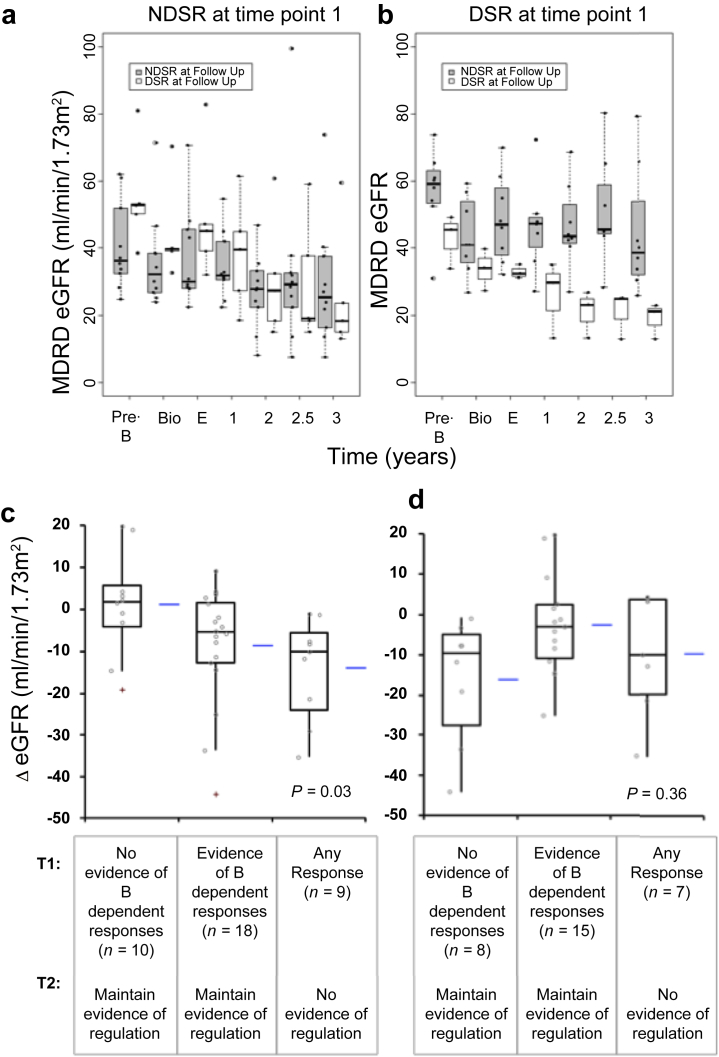
**Associations between patterns on ELISPOT and changes in eGFR in BFC cohort.** Box plots showing the association between the results of ELISPOT assays at time of biopsy and follow-up sample with graft outcome in patients who had viable PBMC samples after thawing at both times (*n* = 27). (**a**) Patients with BFC with NDSR at time of biopsy (*n* = 16), showing stability for those who remained NDSR (*n* = 11) compared with those who became DSR (*n* = 5, *P* = 0.003) and (**b**) patients with BFC with DSR at time of biopsy, showing stable eGFR for those patients who were DSR at time of biopsy (*n* = 11) but converted to NDSR (*n* = 8), compared with progressive decline among those who remained DSR (*n* = 3, *P* = 0.0001). (**c,d**) Box plots show median and IQR, with whiskers representing data within 1.5 the IQR of the upper and lower quartiles, with outliers >1.5 and <3.0 IQR as + and >3 IQR as *. Horizontal lines to right of box plots indicate the mean value. Graphs shows the association between the changes in ELISPOT assays from time point 1 to time point 2 with graft outcome in patients who had 2 viable PBMC samples that could be fully interpreted (i.e., had results from CD8-, CD19-, CD25-, and CD8–CD25-depleted PBMC) (*n* = 37). (**c**) Antidonor responses. Groups correspond to those shown in Table 6 and Supplementary Table S8. Patients at time point 1 with no evidence of B-dependent antidonor responses who maintained evidence of regulated responses at time point 2 had a median ΔeGFR of 1.8 ml/min per 1.73 m^2^ (IQR 6.6) and mean ΔeGFR of 1.2ml/min per 1.73 m^2^ (SD ±12.3). Patients with evidence of B-dependent antidonor responses at time point 1 who maintained evidence of regulated responses at time point 2 had a median ΔeGFR of −5.5 ml/min per 1.73 m^2^ (IQR 12.9) and mean ΔeGFR of −8.6 ml/min per 1.73 m^2^ (SD ±13.7). Finally, patients who had unregulated B-cell–dependent antidonor responses at time point 2 had a median ΔeGFR of −10.1 ml/min per 1.73 m^2^ (IQR 13.7) and mean ΔeGFR of −14 ml/min per 1.73 m^2^ (SD ±12) irrespective of the pattern they had at time point 1. *P* = 0.036. (**d**) Antiviral responses. Groups correspond to those shown in Supplementary Tables S9 and S10. Groups compared by Kruskal-Wallis test. MDRD, Modification of Diet in Renal Disease.

In contrast to when individual time points were considered in isolation, changes in antidonor ELISPOT reactivity in individuals were strongly associated with ΔeGFR (Tables 4 and 5). To assess this further, we estimated generalized linear mixed models separately for those patients from the BFC cohort (with 2 viable ELISPOT samples), who were NDSR (Figure 4a) or DSR (Figure 4b) at time of biopsy. Results showed a statistically significant interaction between the presence of DSR on follow-up samples, and the time of eGFR assessment in both groups (*P* = 0.003 for baseline NDSR, and *P* = 0.0001 for baseline DSR, respectively), indicating that the change in antidonor responses was significantly associated with different patterns of eGFR over time. Remarkably, Figure 4b shows how those patients who had DSR at baseline, but then changed to NDSR, maintained stable function, as opposed to those who remained DSR and showed significant decline. Similarly, patients who developed DSR over follow-up, showed a steeper decline than those who remained DSR negative (Figure 4a).

To assess the whole cohort, and to further address the importance of B-cell phenotype, we performed an analysis of changes in individual patients who had 2 interpretable ELISPOTs. A detailed descriptive analysis is provided in the supplementary file (Results section and Supplementary Table S7) and only a concise interpretation is presented here (Table 6). This analysis showed a significant association between maintenance of nonreactivity or development of regulated donor reactivity at time point 2 and graft stability (Fisher exact *P* = 0.0417). When analyzed by ΔeGFR, the differences between these groups was significant (Figure 4c).

These associations between antidonor ELISPOT pattern changes and eGFR were antigen specific, as an analysis of the responses to control cytomegalovirus and varicella-zoster virus proteins revealed no significant associations with ΔeGFR (Figure 4d), despite the antiviral antigen ELISPOT patterns themselves showing similar changes as those to antidonor proteins (Supplementary Tables S8 and S9).

Altogether, these data support the conclusion that a change from a nonresponsive or regulated antidonor response to unregulated, B-cell–dependent antidonor IFN-γ production is associated with a significant decline in eGFR.

#### Treatment-associated nonresponsiveness/regulation and stability (Table 7)

Changes in antidonor reactivity occurred spontaneously in PROTCL patients, but followed treatment in patients with BFC. To address whether treatment could influence outcome, we selected a homogeneous subgroup of 18 patients with BFC-CAMR, chosen for 3 reasons: (i) they had no tubulitis on biopsy; (ii) they all had ongoing and progressive rises in creatinine, as determined by analysis of reciprocal creatinine plots at the time of first ELISPOT (i.e., this group excluded 5 patients who presented with isolated proteinuria only); and (iii) they were all treated with a protocolized treatment regimen (determined clinically), consisting of addition or optimization of tacrolimus and mycophenolate mofetil followed by i.v. rituximab when oral immunotherapy was thought to have been maximally optimized.

Three had an eGFR <20 ml/min at the time of first ELISPOT, so were excluded. For the remaining 15, 7 became stable (all in “stable” subgroup) and remained stable after treatment (Supplementary Figure S5). All 7 had time point 2 samples showing nonresponsiveness or regulation, and in 5 of 7, it was clear there had been a shift involving loss of B-dependent responses or development of regulated antidonor reactivity or nonresponsiveness. In the 8 patients who showed a continued decline in eGFR (all in the “deteriorating” subgroup), the picture was more complex. Interpretable time point 2 ELISPOTs were available on 6. Three had unregulated B-dependent antidonor activity at time point 2, and 2 of these had clearly lost evidence of regulation that had been present at time point 1, including a patient (ID 635) in whom loss of regulation by B cells followed treatment with rituximab. The 3 remaining were nonresponsive or had regulated antidonor activity at time point 2, but it is notable that 2 of these had infectious complications beyond the time point 2 ELISPOT that necessitated immunosuppression reduction, perhaps confounding an association between their ELISPOT patterns and outcome.

Multivariate logistic regression analysis on those patients with viable time point 1 ELISPOTs in this subgroup was performed. The combined model in this case included age, sex, previous acute rejection, pretransplant dialysis time, HLA Ab and MHC class I polypeptide-related sequence A (MICA) status, C4d on PTC on biopsy, proteinuria, IF/TA, B-cell–dependent antidonor IFN-γ production assay, and treatment with tacrolimus/mycophenolate mofetil/rituximab and generated a receiver operating characteristic curve with 100% sensitivity and 100% specificity (Figure 3c). In this uniformly treated BFC-CAMR subgroup, ELISPOT pattern was a better predictor of outcome than in the whole cohort and was significantly better than HLA Ab status.

### Importance of IL-10 and regulation of IFN-γ in Th-1 CD4+ cells

**Figure 5:**
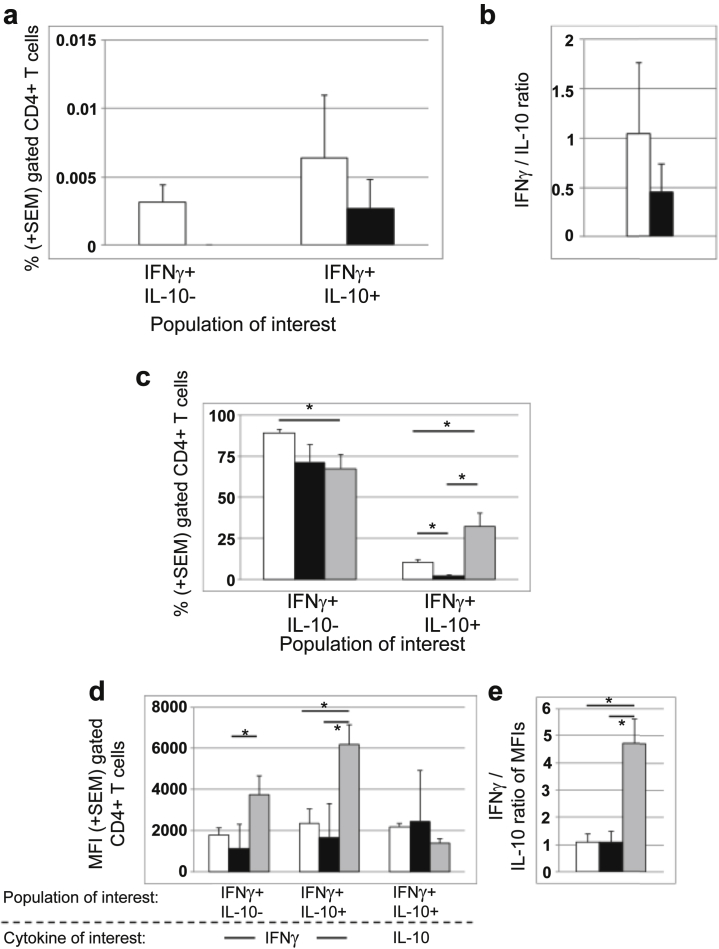
**Flow cytometric analysis of Th-1 cytokine production.** (**a,b**) Donor antigen-specific IFN-γ production by CD4+ T cells: comparison of subgroups according to functional B-cell phenotype on ELISPOT. CD8-depleted PBMCs were stimulated with donor antigen under same conditions as in ELISPOT, then assayed by flow cytometry by using a cytokine capture system. White bars: Samples (*n* = 8) from patients with ELISPOT pattern showing evidence of B-dependent antidonor IFN-γ production (with or without evidence of regulation). Black bars: Samples (*n* = 3) from patients with ELISPOT pattern showing only suppression of antidonor IFN-γ production by B cells with NO evidence of B-dependent responses. (**a**) Shows the percentage of CD4+ cells expressing only IFN-γ (IFN-γ + IL-10−) or coexpressing with IL-10 (IFN-γ + IL-10+). (**b**) Shows the comparison of the percentage of total cells expressing IFN-γ/% total cells expressing IL-10. (**c–e**) Polyclonal stimulation with anti-CD3/anti-CD46 monoclonal antibodies: comparison of subgroups according to functional B-cell phenotype on ELISPOT. White bars: Samples (*n* = 4) in which antidonor-specific ELISPOT showed only suppression of antidonor IFN-γ production by B cells with NO evidence of B-dependent responses. Black bars: Samples (*n* = 8) in which antidonor-specific ELISPOT showed evidence of a regulated B-dependent antidonor response. Gray bars: Samples (*n* = 4) in which antidonor-specific ELISPOT showed evidence of an unregulated B-dependent antidonor response. (**c**) Percentage of CD4+ cells staining for IFN-γ alone (IFN-γ + IL-10−) compared with cells staining for both (IFNγ + IL-10+). (**d**) Median fluorescence intensity of staining for IFN-γ or IL-10 in the single-positive (IFNγ + IL-10−) or double-positive (IFNγ + IL-10+) CD4+ populations as indicated. (**e**) Ratio of mean fluorescence intensity of IFN-γ staining to IL-10 staining in the double-positive (IFN-γ + IL-10+) population in (**d**). *P < 0.05 by Mann-Whitney *U* test.

As described in detail in the supplementary materials, experiments to assess whether functional Breg (increase in spot count of ≥20% when CD19+ cells depleted) activity involved IL-10 secretion indicated that there was an additional source of IL-10 in ELISPOTs besides B cells. To explore whether T cells might themselves be making IL-10,^10^, ^11^ we selected samples with sufficient cells available, stimulated PBMC with donor material, and assessed IL-10 and IFN-γ single or coexpression by CD4^+^ cells (Figure 5a and 5b). In samples showing evidence of B-cell regulation without any B-dependent antidonor responses on ELISPOT (*n* = 3), all CD4^+^ T cells expressing IFN-γ also expressed IL-10, whereas cells expressing IFN-γ alone were evident only in samples in which there was evidence of a B-dependent antidonor response (*n* = 9). The frequencies of cytokine-positive antidonor CD4^+^ T cells revealed by these analyses were consistent with those seen in ELISPOT assays. These data suggest that “pure” B-cell regulation in ELISPOT associates with IL-10 expression by IFN-γ–producing CD4^+^ T cells.

A failure of this autocrine mechanism, resulting in Th-1 cells that produce large amounts of IFN-γ, with a proportional reduction in IL-10 coexpression, has been associated with active rheumatoid arthritis (RA).^12^ We polyclonally stimulated all samples from which we had sufficient PBMCs (*n* = 16) to assess if such cells were present (Figure 5c–5e). CD4^+^ cells from 4 samples produced significantly more IFN-γ than IL-10, consistent with the proinflammatory phenotype seen in patients with RA^12^; these samples had a high frequency (>20%) of double-positive T cells expressing both IFN-γ and IL-10. The other 12 samples had low frequencies of double-positive CD4+ T cells that secreted as much IL-10 as IFN-γ (Figure 5c–5e).

Importantly, all 4 samples containing the highly inflammatory Th-1 cells showed an identical pattern on antidonor ELISPOT assay (B-cell–dependent reactivity without any evidence of regulation by B or T cells), whereas the others showed either no evidence of B-dependency, or regulated B-dependent antidonor activity and appeared to respond similarly to polyclonal stimulation (Figure 5c–5e).

All these data suggest that regulation by B cells in antidonor ELISPOT involves activation of an autocrine IL-10 regulatory pathway, to tip the balance from IFN-γ expression by Th-1 cells to preferential IL-10 production, and that a failure of this regulatory pathway associates with unregulated B-cell–dependent antidonor responses.

## Discussion

The association between DSA and graft failure is well established^5^, ^13^, ^14^; however, the significant variability in clinical phenotype associated with DSA^15^, ^16^, ^17^, ^18^ is difficult to explain. Differences in the functional characteristics of DSA, such as the subclass of IgG^19^ or the ability to fix complement,^20^ offer a potential explanation. However, other factors associated with the presence of DSA might influence the progression of pathology, rate of functional deterioration, and timing of eventual graft failure.

HLA Abs are a marker of B-cell activation, which is a T-cell–dependent process involving cognate interactions between B and T cells, so it therefore follows that DSAs are markers of “indirect” CD4^+^ T-cell sensitization to donor antigens. “Indirect” in this context refers to a specific pathway of allorecognition in which graft antigens are processed into peptide fragments and presented on recipient HLA class II molecules by professional antigen-presenting cells. Indirect responses to mismatched donor HLA have been associated with graft dysfunction and chronic rejection in both renal^21^, ^22^, ^23^, ^24^, ^25^ and cardiac allografts.^26^, ^27^ Our previous report confirmed that B cells acted as antigen-presenting cells for indirect alloresponses in patients with CAMR,^7^ and also described the complexities of antidonor reactivity, such that a significant proportion had evidence of active regulation of their antidonor responses. This report addresses the hypothesis that the activity of these cellular immune responses is one of the significant “other factors” that influence the progression of graft dysfunction.

We confirmed, as reported by others, that DSA,^28^ peritubular capillaritis,^29^ and IF/TA^30^ on biopsy, along with proteinuria, were all associated with graft failure. Of these traditional factors, only DSA and proteinuria were associated with ΔeGFR, although with relatively poor sensitivity and predictive value. With regard to ELISPOT patterns, B-cell–dependent antidonor IFN-γ production was the factor most strongly correlated with graft dysfunction in the PROTCL subgroup. Within the BFC subgroup, correlations between B-cell–dependent antidonor reactivity at time point 1 and ΔeGFR were weaker; in this subgroup, time point 2 samples appeared to have a stronger association with outcome, as they did in analysis of the whole cohort. Most impressively, this was evident in patients in whom antidonor reactivity changed from nonresponsiveness or a regulated response at time point 1, to an unregulated B-cell–dependent response at time point 2: these patients showed the greatest loss of GFR. Conversely, those with B-cell–dependent antidonor reactivity who became nonresponsive or developed evidence of T- or B-cell regulation, appeared to stabilize and maintain GFR over the course of the study. This was seen clearly as a “treatment effect” in a subset of selected patients with BFC-CAMR who received an optimized treatment protocol for “creeping creatinine,” in whom the predictive value of the time point 1 ELISPOT pattern was enhanced and was better than HLA Ab status at predicting ΔeGFR. We believe the main reason why univariate associations were not seen in subgroup analyses at all time points was the small number of patients in our analysis, compounded by the fact that ELISPOT interpretations were complex, so that when they were defined according to the patterns revealed by sequential depletion of CD25 /CD19 cells, associations were mostly evident only in analysis of the whole group.

Nevertheless, multivariate testing indicated the ELISPOT pattern to be an independent factor that predicted changes in eGFR in both PROTCL and BFC groups, as demonstrated by the superior AUCs obtained from our prediction modeling when ELISPOT patterns were included. Our statistical methodology was chosen so we could estimate the predictive accuracy of all variables irrespective of their *P* values; where individual variables were selected for testing, elastic net regression, tuned via cross-validation, was used because of the small sample sizes.

These data provide the first potential explanation for the findings of Wiebe *et al.*,^31^ who described patients with DSA, who were compliant with immunosuppressive medication and had stable graft function, suggesting that maintaining “control” of T and B cells with conventional immunosuppression is sufficient to achieve stability of function in some patients with DSA. They are also compatible with the recent report from Shabir *et al.*,^32^ who described a link between stable graft function and preserved peripheral transitional B-cell proportions, even in a small number of patients who developed de novo DSA, although we were unable to correlate a specific surface B-cell phenotype with the functional phenotype revealed by ELISPOT.^7^ Our results also provide a basis for understanding the reports of how enhanced immunosuppression can stabilize function in patients with CAMR. Theruvath *et al.*^33^ reported 12-month stabilization of kidney function in 3 of 4 patients with CAMR after transfer onto tacrolimus and mycophenolate mofetil and a short course of prednisolone. In addition, several studies have reported successful stabilization after B-cell depletional therapy,^34^, ^35^, ^36^ supporting the hypothesis that underlying cellular responses are contributing to functional deterioration in these patients.

Eight of our patients received rituximab, but no definitive conclusions from these small numbers can be made. However, as well as patient 635 (highlighted previously in this article), who lost evidence of B-cell regulation after rituximab, another patient (326) stabilized eGFR after rituximab in association with development of a regulated antidonor response (there was no change in the median fluorescence intensity of DSA assessed by Luminex [xMAP assay (Thermofisher, California, USA)] in either patient [Supplementary Table S1c and c]). Both cases suggest that response to rituximab might be informed by knowledge of ELISPOT patterns pretreatment.

It is important to state, as we have before,^7^ that we were not able to purify putative regulatory T or B cells from patients due to lack of cells. Therefore, the conclusions made are based on indirect evidence of regulation, on depleting specific lymphocyte subsets, rather than a direct demonstration of suppressive activity from purified, and then *in vitro* assessed cell subpopulations. We presented our flow cytometric analysis of these samples in our earlier article last year, and found no associations between the proportions of B-cell subsets, including transitional or naïve cells and ELISPOT patterns or outcome,^7^ although we acknowledge that others working in this field have shown associations between rejection and particular B-cell subsets, including transitional B cells.^32^, ^37^, ^38^ In addition, the associations reported in this article need to be validated in different cohorts of patients, ideally with work to assess the reproducibility of the assays within patients and to more carefully document how patterns change over time.

Importantly, for the first time, we have attempted to link antigen-specific indirect alloresponses with mechanisms by which IFN-γ production is regulated in Th-1 CD4^+^ T cells. Physiological regulation of these cells, by IL-10, is known to be essential, as unchecked IFN-γ production results in severe tissue damage, as illustrated by the responses to *Listeria* or *Trypanosoma* disease in IL-10–deficient mice.^10^ Although multiple cell types can make IL-10, that made by the Th-1 cells themselves^39^ is the major *in vivo* source^40^ and critical to providing regulatory feedback, via antigen-presenting cells and T cells themselves, to prevent inappropriate Th-1–driven immunopathology.^41^ Aligning with this model, we showed that an anti–IL-10 antibody caused significant increase in the frequency of IFN-γ producing spots in ELISPOT, even in the absence of B cells, and additionally, that in CD8-depleted samples showing evidence of only a B-regulated response, Th-1 cells stimulated by donor antigen only made IFN-γ in the context of coexpression with IL-10. We have not attempted to assess the predominant source of IL-10 in our assays, and our data cannot exclude an important role for B-cell–derived IL-10 in regulation of antidonor alloresponses, as others working in this area have shown.^37^, ^42^, ^43^

Abnormalities of IL-10 switching mediated by CD46 signaling have been associated with excessive IFN-γ production by Th1 cells from synovial fluid of patients with RA.^12^ T cells from patients with active RA fail to shut down IFN-γ production on CD46-activation, and, perhaps counterintuitively, have high proportions of IFN-γ + IL-10 + double-positive cells, but these express a very large amount of IFN-γ compared with T cells from healthy individuals.^10^, ^12^ Using an *in vitro* system involving polyclonal stimulation through CD46,^44^ we found cells similar to those described in patients with RA in 4 of 16 samples, all of which demonstrated the same pattern of antidonor response (unregulated B-cell–dependent reactivity), whereas the other 12 samples showing evidence of regulation resembled responses seen in healthy controls.^12^

These data imply, for the first time, that functional suppression by B cells in ELISPOT activates this IL-10 autocrine pathway of regulation to restrict IFN-γ production by Th-1 cells. Moreover, inability to switch off IFN-γ production via this regulatory mechanism associates with a specific pattern of unregulated antidonor response that, as we have demonstrated here, is associated with a greater loss of renal function over time. Better understanding of this regulatory mechanism may lead to the development of more sophisticated treatments for chronic rejection.

In summary, our analysis of the cell-mediated IFN-γ production by PBMCs against donor antigens has generated 2 important and novel findings. First, a significant association between patterns of antidonor ELISPOT reactivity and eGFR outcomes, with evidence that treatment to influence antidonor responses can affect patient outcomes: these findings support the hypothesis we set out to test, that cell-mediated immunity has a strong influence on deterioration in patients with CAMR. Second, we have defined a novel link between B-cell regulation of IFN-γ production and an IL-10–dependent autocrine mechanism regulating Th-1 CD4^+^ T cells, with the implication that manipulation of this mechanism might significantly affect the evolution of indirect alloresponses, and ultimately, on long-term allograft survival.

## Materials and Methods

Methodology is exactly as described in a previous report.^7^ Full details are given in the Methods section of the Supplementary Materials and Methods. Brief descriptions are given here.

### Experimental design and recruitment

The study was performed as part of a large observational study looking at the importance of HLA antibodies posttransplantation, the protocol of which was approved by the Hammersmith, Queen Charlotte’s, and Chelsea and Acton Hospitals Research Ethics Committees (2002/6452) and conformed to the 1964 Declaration of Helsinki and subsequent amendments. All participants gave written informed consent before inclusion. Calculation of eGFR, ΔeGFR, and details of blood collection and processing is described in the Methods section of the supplementary materials.

### ELISPOT assay

IFN-γ ELISPOT plates (Mabtech AB, Nacka, Sweden) precoated with primary IFN-γ Ab were blocked for 2 hours with “complete medium” (AIM-V medium/10% human AB serum from Life Technologies [Paisley, UK]) before addition of 4 x 10^5^ responder PBMCs per well in 100 μl complete medium with donor antigens. PBMCs were prepared according to standard laboratory protocols. Controls and source of donor antigen are described in detail in the Methods section of the supplementary materials.

### Statistical analysis

Statistical analyses were performed by using R.^45^ Two-sided tests were used throughout, and a *P* < 0.05 was considered statistically significant in univariate statistical testing. Group differences were assessed by using Fisher χ^2^ test for categorical variables, Wilcoxon rank-sum (Mann-Whitney) or Kruskal-Wallis tests for non-normally distributed continuous variables, and *t*-test for normally distributed continuous variables (for paired or unpaired samples as appropriate). For prediction analysis, we estimated generalized linear models for predefined groups of predictive variables. Only baseline variables were added to generalized linear prediction models, and thus fixed effects only models were fitted. The estimated predicted probability of outcome was then used to build a receiver operating characteristic curve, and estimate the AUC, sensitivity, and specificity.^46^ To obtain the optimal combination of predictors of outcome, we used elastic net models. Elastic net is a regularized regression method in which a penalty is imposed on the regression coefficients, which is a combination of the penalties used in lasso and ridge regression. Elastic net enables selection of predictors (unlike ridge regression, which would moderate coefficients but not make them 0), and can handle and select groups of correlated predictors (unlike lasso, which would select only 1 of a group of correlated predictors, and drop the rest).^47^

For the analysis of patterns of change in eGFR over time in patients with and without baseline DSR, we fitted linear mixed-effects models. Separate models were fitted for patients with and without baseline DSR. The model included an intercept, main effects, and interaction between study time point (from prebiopsy to 3-years’ follow-up) and DSR status at time point 2 as fixed effects, as well as a random intercept for the subject.

## Disclosure

All the authors declared no competing interests.

## Figures and Tables

**Table 1:** Associations between clinical and biopsy features, DSA and patient outcomes

Clinical variable	Result in whole cohort	Number of biopsies/samples		*P*^a^	Number of biopsies/samples		*P*^a^
		Graft failure	No graft failure		Deteriorating eGFR (≤median)	Stable eGFR (>median)	
PCR >50 at time of biopsy	Yes (*n =* 25)	10	15	**0.002**	17	8	**0.03**
	No (*n =* 27)	1	26		10	17	
Biopsy subgroup	BFC (*n =* 37)	11	26	**0.02**	19	18	1
	PROTCL (*n =* 15)	0	15		8	7	
Gross biopsy features	AMR (*n =* 45)	11	34	0.3	25	20	0.24
	Control (*n =* 7)	0	7		2	5	
Tubulitis	Positive (*n =* 4)	2	2	0.2	3	1	0.6
	Negative (*n =* 48)	9	39		24	24	
C4d (PTC)	Positive (*n =* 26)	8	18	0.1	16	10	0.27
	Negative (*n =* 26)	3	23		11	15	
C4d (g)	Positive (*n =* 30)	9	21	0.09	17	13	0.58
	Negative (*n =* 22)	2	20		10	12	
G score	≥1 (*n =* 21)	6	15	0.3	14	7	0.1
	0 (*n =* 31)	5	26		13	18	
PTC score	≥1 (*n =* 15)	7	8	**0.008**	8	7	1
	0 (*n =* 37)	4	33		19	18	
CG score	≥1 (*n =* 20)	7	13	0.08	10	10	1
	0 (*n =* 32)	4	28		17	15	
CV score	≥1 (*n =* 22)	6	16	0.5	13	9	0.57
	0 (*n =* 29)	5	24		14	15	
% Median IF/TA score		30	15	**<0.05**^b^	20	15	>0.05
DSA time point 1	>1000 (*n =* 18)	7	11	**0.03**	12	6	0.15
	0 or <1000 (*n =* 34)	4	30		15	19	
DSA time point 2	>1000 (*n =* 18)	7	11	**0.04**	11	7	0.39
	0 or <1000 (*n =* 32)	4	28		15	17	
DSA overall^c^	>1000 (*n =* 20)	7	13	0.08	12	8	0.4
	0 or <1000 (*n =* 32)	4	28		15	17	

Proteinuria: Graft failure – Sensitivity: 10/11 = 91%; PPV: 10/25 = 40%; NPV: 26/27 = 96%; Specificity: 26/41 = 63%.

Proteinuria: Deteriorating function – Sensitivity: 17/27 = 63%; PPV 17/25 = 68%; NPV 17/27 = 63%; Specificity 68%.

PTC score: Graft failure – Sensitivity: 7/11 = 64%; PPV: 7/15 = 67%; NPV: 33/37 = 89%; Specificity: 33/41 = 80%.

DSA >1000 time point 1: Graft failure – Sensitivity: 7/11 = 64%; PPV: 7/18 = 39%; NPV: 30/34 = 88%; Specificity: 30/41 = 77%.

DSA >1000 time point 2: Graft failure – Sensitivity: 7/11 = 64%; PPV: 7/18 = 39%: NPV: 28/32 = 88%; Specificity: 28/39 = 72%.

Bold *P* values are statistically significant.

AMR, antibody-mediated rejection; BFC, for-cause biopsy; DSA, donor-specific antibody; eGFR, estimated glomerular filtration rate; NPV, negative predictive value; PCR, protein-to-creatinine ratio; PPV, positive predictive value; PROTCL, protocol; PTC, peritubular capillary; g, glomerulitis; cg/cv, BANFF chronic glomerulopathy and vascular scores; IF/TA, interstitial fibrosis/tubular atrophy.

a Fisher exact test.

b Mann-Whitney *U* test.

c DSA at either time point 1 or 2 or both.

**Table 2:** ELISPOT patterns, classified by response to donor antigens when CD8+ cells depleted as DSR or NDSR and, for the latter by the response after depletion of CD25+ cells (“Treg”) or CD19+ cells (Breg)

ELISPOT patterns				Interpretation based on reactivity of CD8-depleted PBMC (DSR) versus nonreactivity (NDSR)^a^		Interpretation based on B-cell phenotype	
CD25 present		CD25 depleted					
CD8-deplete	CD8- and CD19-deplete	CD8-deplete	CD8 and CD19-deplete				
−	−	−	−	NDSR	No regulation	No regulation	No response

−	+^b^	−	−		Breg	Breg: only when CD25+ cells present	Regulated antidonor response without evidence of B-dependency

−	+	−	+		Breg: when CD25+ cells present or absent		

−	−	−	+	Treg, Breg	Breg: only when CD25+ cells absent

−	+	+	−	Breg when CD25 present BUT Bdep when CD25 absent	B-dependent antidonor response with evidence of regulation

−	−	+	−	Treg		Bdep: only when CD25+ cells absent
+	−	−	+	DSR	Bdep	Bdep when CD25 present, Breg when CD25+ cells absent
+	−	+	−			Bdep: when CD25+ cells present and absent	Unregulated B-dependent antidonor response
+	−	−	−			Bdep: only when CD25+ cells present	

An alternative way to interpret these patterns is by the functional B-cell phenotype in the presence or of CD25+ cells. Note that some samples defined in Shiu *et al.*^7^ as DSR Bdep showed evidence of Breg activity in absence of CD25+ cells.

Bdep, decrease in spot count of ≥20% when CD19+ cells depleted; Breg, increase in spot count of ≥20% when CD19+ cells depleted; DSR, donor-specific reactivity; ELISPOT, enzyme-linked immunosorbent spot; NDSR, no donor-specific reactivity; PBMC, peripheral blood mononuclear cell; Treg, increase in spot count of ≥20% when CD25+ cells depleted.

a As used in Shiu *et al*.^7^

b Threshold for defining positive antidonor interferon-γ production was 25 or more spots per million CD4+ cells on donor antigen plate compared with background. Therefore, “+” = spot count ≥25; “−” =spot count <25.

**Table 3:** Number of samples ELISPOT patterns interpreted by B-cell phenotype in PROTCL and BFC by time

Interpretation based on B-cell phenotype				Number of ELISPOTs showing the defined pattern					
				PROTCL		BFC		Total	
				Time point 1	Time point 2	Time point 1	Time point 2	Time point 1	Time point 2
No evidence of B-dependent antidonor response	No response		No regulation	3	1	5	11	8	12

	Evidence of regulation	Regulated antidonor response without evidence of B-dependency	Breg: only when CD25+ cells present	2	2^a^	0	1	5	7
			Breg: when CD25+ cells present and absent	0	2	2	0		
Breg: only when CD25+ cells absent			0	1	1	1			
Evidence of B-dependent antidonor response		B-dependent antidonor response with evidence of regulation	Breg when CD25 present BUT Bdep when CD25 absent,	1	0	5^b^	3^c^	12	13

			Bdep: only when CD25+ cells absent	1	3	4	3		
	Bdep when CD25 present, Breg when CD25+ cells absent	0	1	1	3

No evidence of regulation	Unregulated B-dependent antidonor response	Bdep: when CD25+ cells present and absent	3	0	7	3	13	11
		Bdep: only when CD25+ cells present	2	4	1	4		
Not done / Not viable / Not interpretable^d^				2 ND 1 NDSR	1 NDSR	5 ND 3 NDSR 3 DSR	5 ND 3 NDSR	14	9

Several other viable samples at later time points were collected and analyzed and included in Shiu *et al.*^7^ but are not considered further here.

Bdep, decrease in spot count of ≥20% when CD19+ cells depleted; BFC, for-cause biopsy; DSR, donor-specific reactivity; Breg, increase in spot count of ≥20% when CD19+ cells depleted; ELISPOT, enzyme-linked immunosorbent spot; IFN-γ, interferon-γ; NDSR, no donor-specific reactivity; PBMC, peripheral blood mononuclear cells; PROTCL, protocol.

a One of these samples was DSR (i.e., IFN-γ produced by CD8-depleted PBMCs but spot count increased by >20% with B-cell depletion. Therefore, there were 7 DSR and 8 NDSR in PROTCL at time point 2.

b Three of these samples were DSR (i.e., IFN-γ produced by CD8-depleted PBMCs but spot count increased by >20% with B-cell depletion. All 3 also showed increases >20% with depletion of CD25+ cells followed by reduction (>20%) in spot count when CD19+ cells additionally depleted. Therefore, there were 14 DSR and 18 NDSR in BFC at time point 1.

c One of these samples was DSR (i.e., IFN-γ produced by CD8-depleted PBMCs but spot count increased by >20% with B-cell depletion. Both also showed increases >20% with depletion of CD25+ cells followed by reduction (>20%) in spot count when CD19+ cells additionally depleted. Therefore, there were 11 DSR and 21 NDSR in BFC at time point 2.

d In some samples, it was not possible to perform all 4 depletion combinations to enable interpretation based on B-cell phenotype, but classification as DSR/NDSR was possible.

**Table 4:** Association between antidonor reactivity (DSR/NDSR) and patient outcomes in whole cohort

ELISPOT variable	ELISPOT pattern	Number of samples		*P*^a^	Number of samples		*P*^a^
		Graft failure	No graft failure		Deteriorating eGFR (≤median)	Stable eGFR (>median)	
Time point 1	DSR (*n =* 20)	3	17	0.47	11	9	0.80
	NDSR (*n =* 25)	7	18		12	13	
Time point 2	DSR (*n =* 17)	5	12	0.23	11	6	0.08
	NDSR (*n =* 30)	3	27		11	19	
Change to or maintenance of^b^:	DSR (*n =* 15)	5	10	0.08	10	5	**0.05**
	NDSR (*n =* 25)	2	23		8	17	

Bold *P* values are those that are statistically significant.

DSR, donor-specific reactivity; eGFR, estimated glomerular filtration rate; ELISPOT, enzyme-linked immunosorbent spot; NDSR, no donor-specific reactivity.

a Fisher exact test.

b In paired samples only (i.e., samples in which ELISPOTS available at both time points); in relation to graft failure, only 7 of the 11 with graft failure had ELISPOTS at both time points.

**Table 5:** Association between antidonor reactivity based on functional B-cell phenotype and patient outcomes in whole cohort

ELISPOT variable	ELISPOT pattern	Number of samples		*P*^a^	Number of samples		*P*^a^
		Graft failure	No graft failure		Deteriorating eGFR (≤median)	Stable eGFR (>median)	
Time point 1	No evidence of B-dependence (*n =* 13)	2	11	0.69	4	9	0.09
	Evidence of B-dependence (*n =* 25)	6	19		16	9	
Time point 2	No evidence of B-dependence (*n =* 19)	1	18	0.11	5	14	**0.03**
	Evidence of B-dependence (*n =* 24)	6	18		15	9	
Change to or maintenance of:^b^	No evidence of B-dependence (*n =* 16)	1	15	0.20	4	12	**0.04**
	Evidence of B-dependence (*n =* 21)	5	16		13	8	

Bold *P* values are those that are statistically significant.

eGFR, estimated glomerular filtration rate; ELISPOT, enzyme-linked immunosorbent spot.

a Fisher exact test.

b In paired samples only (i.e., samples in which ELISPOTs available at both time points).

**Table 6:** Dynamic changes in antidonor ELISPOT patterns and association with outcome

Interpretation based on B-cell phenotype			Time point 2^a^			
			No response	Evidence of regulation		No evidence of regulation
				Regulated antidonor response without evidence of B-dependency	B-dependent antidonor response with evidence of regulation	Unregulated B-dependent antidonor response
Time point 1^b^	No evidence of B-dependent antidonor response	No response	10 patients: 8 stable, 2 deteriorating ΔeGFR^c^ 1.79 (IQR 6.63)			9 patients: 2 stable, 7 deteriorating ΔeGFR −10.1 (IQR 13.7)^d^
		Regulated antidonor response without evidence of B-dependency				

	Evidence of B-dependent antidonor response	B-dependent antidonor response with evidence of regulation	18 patients: 10 stable, 8 deteriorating ΔeGFR −5.54 (IQR 12.9)			
Unregulated B-dependent antidonor response					

Refer to Supplementary Table S7 for full details of all patients.

eGFR, estimated glomerular filtration rate; ELISPOT, enzyme-linked immunosorbent spot; IQR, interquartile range.

a Six additional patients with time point 1 samples had time point 2 samples that were either not done or not fully interpretable, so they are not included in this analysis; 3 patients had neither time point 1 or 2 samples that could be interpreted by B-cell phenotype so they are not included here.

b Six additional patients with time point 2 samples had time point 1 samples that were either not done or not fully interpretable, so they are not included in this analysis.

c eGFR in ml/min per 1.73m^2^.

d Comparison of stable and deteriorating patients in each group: *P* = 0.047 Fisher Exact Probability 3 x 2 test.

**Table 7:** Summary details of demographics, biopsy, and immunosuppressive treatment of optimized CAMR patients: details of the 18 patients treated with optimization (Tac/MMF ± rituximab) for deteriorating creatinine

Patient ID	PCR >50	Changes in treatment postbiopsy	B phenotype in ELISPOT T1	B phenotype in ELISPOT T2	Renal outcome at 3 yr	Adverse events
165	No	CsA to Tac switch	Regulated Bdep	Breg	Stable GFR, no proteinuria	0
326	Yes	MMF and rituximab	Bdep - no reg	Regulated Bdep	Stable GFR, ongoing proteinuria	0
392	Yes	CsA to Tac switch	Regulated Bdep	NR	Graft loss, 2 yr after biopsy	Staph sepsis/joint infection, 7 mo after switch to Tac
397	Yes	MMF	Bdep - no reg	Regulated Bdep	Stable GFR, proteinuria resolved	0
399	No	Rituximab	NR	NR	Continued deterioration, no proteinuria	0
438	Yes	MMF	Bdep - no reg	Regulated Bdep	Stable GFR, continued proteinuria	0
635	Yes	Rituximab	Breg	Bdep - no reg	Graft loss, 22 mo after biopsy	0
739	No	CsA to Tac switch	NR	NR	Stable GFR, no proteinuria	Recurrent UTI. No serious infections.
807 (<20)	Yes	CsA to Tac switch	-	-	Graft loss at 15 mo after biopsy	0
835	Yes	CsA to Tac, Aza to MMF switch	Breg	Bdep - no reg	Graft loss at 12 mo postbiopsy. Rituximab (9 mo postbiopsy)	0
841	No	CsA to Tac switch	Bdep - no reg	NR	Stable GFR, no proteinuria	0
861	Yes	CsA to Tac, Aza to MMF switch, rituximab	Nonviable	Bdep - no reg	Continued deterioration, no proteinuria	Nausea and vomiting 1 mo after rituximab. No cause found. Settled spontaneously.
965	No	Optimized Tac, MMF levels	Nonviable	NR	Stable GFR, no proteinuria	0
1364	Yes	Rituximab	NR	NR	Continued deterioration, continued proteinuria	*Aspergillus* and *Stenotrophomonas* lung infection 2 wk after first dose of rituximab. Not given second dose.
1404	Yes	MMF. Steroids (3 mo postbiopsy) Rituximab (8 mo postbiopsy)	Regulated Bdep	Nonviable	Graft loss at 22 mo postbiopsy	*Pseudomonas* and *Klebsiella* soft tissue infection (orbital cellulitis) 13 mo postbiopsy (5 mo after rituximab).
2002	Yes	Rituximab	Bdep - no reg	Not done	Graft loss at 7 mo postbiopsy/rituximab	0
2006 (<20)	Yes	Tac, MMF, rituximab	-	-	Graft loss at 9 mo postbiopsy	0
2010 (<20)	Yes	CsA to Tac switch	-	-	Graft loss at 16 mo postbiopsy	0

Rows highlighted in gray are the patients who stabilized their renal function after treatment. Refer to Supplementary Figure S6 for the plots of Modification of Diet in Renal Disease and change in estimated GFR. Deteriorating GFR at time of biopsy confirmed by analysis of 1/creatinine plot. Stability and continued deterioration after 3 years were confirmed also on analysis of 1/creatinine plots.

Bdep - no reg, unregulated B-dependent antidonor response; Breg, regulated antidonor response without evidence of B-dependency; CAMR, chronic antibody-mediated rejection; ELISPOT, enzyme-linked immunosorbent spot; GFR, glomerular filtration rate; MMF, mycophenolate mofetil; NR, nonresponsive; PCR, protein-to-creatinine ratio; regulated Bdep, B-dependent antidonor response with evidence of regulation; T1, time point 1; T2, time point 2; CsA, ciclosporin A; Tac, tacrolimus; Aza, azathioprine; UTI, urinary tract infection. ELISPOT responses for patients 807, 2006, 2010 are not provided at T1 or T2 as they had eGFR <20 and were therefore excluded from analysis.

## Supplementary Material

Supplementary Methods

Figure S1 Lack of association between PTC score and ΔeGFR and between IF/TA and ΔeGFR over the course of the study in combined PROTCL and BFC group. (**A**) Box plots show median with IQR with whiskers showing upper and lower limits of ΔeGFR. Combined group includes all PROTCL and BFC patients, except 9 patients with BFC who either had missing follow-up data (*n* = 2) or eGFR ≤20 ml/min per 1.73 m^2^ at time of biopsy (*n* = 7). Patients with PTC score <1 have median ΔeGFR of −6.85 ml/min per 1.73 m^2^ (IQR 12.4) and mean ΔeGFR of −8.7 ml/min per 1.73 m^2^ (SD ±13.1). Patients with PTC score ≥1 have median ΔeGFR of −8.0 ml/min per 1.73 m^2^ (IQR 16.7) and mean ΔeGFR of −7.2 ml/min per 1.73 m^2^ (SD ±15.1). *Mann-Whitney *U* test. (**B**) Graph shows lack of correlation between IF/TA % on biopsy and ΔeGFR for each of the patients (*n* = 52) included in this analysis.

Figure S2 Association between DSA and ΔeGFR over the course of the study in combined cohort. Box plots show median with IQR with whiskers showing upper and lower limits of ΔeGFR. Combined group includes all PROTCL and BFC patients, except 9 patients with BFC who either had missing follow-up data (*n* = 2), or eGFR ≤20 ml/min 1.73 m^2^ at time of biopsy (*n* = 7). Time point 1: Patients with No DSA or DSA with cumulative mean fluorescence intensity of <1000 (*n* = 35) have median ΔeGFR of −3.19 ml/min per 1.73 m^2^ (IQR 12.3) and mean ΔeGFR of −6.24 ml/min 1.73 m^2^ (SD ±13.4). Patients with DSA with cumulative mean fluorescence intensity >1000 (*n* = 17) have median ΔeGFR of −11.4 ml/min per 1.73 m^2^ (IQR 10.7) and mean ΔeGFR of −12.4 ml/min per 1.73 m^2^ (SD ±13.3). Time point 2: Patients with No DSA or DSA with cumulative mean fluorescence intensity of <1000 (*n* = 34) have median ΔeGFR of −3.69 ml/min per 1.73 m^2^ (IQR 12.4) and mean ΔeGFR of −5.68 ml/min/1.73 m^2^ (SD ±11.9). Patients with DSA with cumulative mean fluorescence intensity >1000 have median ΔeGFR of −9.9 ml/min per 1.73 m^2^ (IQR 15) and mean ΔeGFR of −13.0 ml/min per 1.73 m^2^ (SD ±16). **Mann-Whitney *U* test.

Figure S3 Box plots show median with IQR with whiskers showing upper and lower limits of ΔeGFR in PROTCL (**A**) and BFC (**B**) subgroups. Time point 1: PROTCL patients with DSR have median ΔeGFR of −8.34 ml/min per 1.73 m^2^ (IQR 17.4) and mean ΔeGFR of −17.3 ml/min per 1.73 m^2^ (SD ±17.4), compared with those with NDSR who have a median ΔeGFR of −2.03 ml/min per 1.73 m^2^ (IQR 6.2) and mean ΔeGFR of −2.38 ml/min per 1.73 m^2^ (SD ±4.9). Patients with BFC with DSR have median ΔeGFR of −6.4 ml/min per 1.73 m^2^ (IQR 11.7) and mean ΔeGFR of −7.1 ml/min per 1.73 m^2^ (SD ±10.7), compared with those with NDSR who have median ΔeGFR of –9.9 ml/min per 1.73 m^2^ (IQR 20.1) and mean ΔeGFR of −10.3 ml/min per 1.73 m^2^ (SD ±17.1). Actual *P* = 0.46. Time point 2: PROTCL patients with DSR have median ΔeGFR of −4.5 ml/min per 1.73 m^2^ (IQR 7.2) and mean ΔeGFR of −4.6 ml/min per 1.73 m^2^ (SD ±5.4), compared to those with NDSR who have median ΔeGFR of −4.2 ml/min per 1.73 m^2^ (IQR 15.8) and mean ΔeGFR of −10.9 ml/min per 1.73 m^2^ (SD ±15.5). Patients with BFC with DSR have median ΔeGFR of −12.7ml/min per 1.73 m^2^ (IQR 13.5) and mean ΔeGFR of −13.5 ml/min per 1.73 m^2^ (SD ±14.6), compared with those with NDSR who have median ΔeGFR of –2.5 ml/min per 1.73 m^2^ (IQR 12.3) and mean ΔeGFR of −2.4 ml/min per 1.73 m^2^ (SD ±12.1). Actual *P* = 0.01. **Mann-Whitney *U* test.

Figure S4 Box plots show median with IQR with whiskers showing upper and lower limits of ΔeGFR in PROTCL (upper panel) and BFC (lower panel) subgroups. Time point 1: PROTCL patients with no evidence of B-dependent antidonor responses have median ΔeGFR of +1.5 ml/min per 1.73 m^2^ (IQR 3) and mean ΔeGFR of −0.54 ml/min per 1.73 m^2^ (SD ±2.6), compared with those with evidence of B-dependent antidonor reactivity, who have a median ΔeGFR of −7.7 ml/min per 1.73 m^2^ (IQR 11.7) and mean ΔeGFR of −13.8 ml/min per 1.73 m^2^ (SD ±15.4). Patients with BFC with no evidence of B-dependent antidonor responses have median ΔeGFR of −11.1 ml/min per 1.73 m^2^ (IQR 27.6) and mean ΔeGFR of −6.9 ml/min per 1.73 m^2^ (SD ±19.7), compared with those with evidence of B-dependent antidonor reactivity, who have median ΔeGFR of –9.7 ml/min per 1.73 m^2^ (IQR 12) and mean ΔeGFR of −10.3 ml/min per 1.73 m^2^ (SD ±12.4). Actual *P* = 0.88 by Mann-Whitney *U*. Time point 2: PROTCL patients with no evidence of B-dependent antidonor responses have median ΔeGFR of −1.7 ml/min per 1.73 m^2^ (IQR 3.9) and mean ΔeGFR of −7.7 ml/min per 1.73 m^2^ (SD ±18), compared with those with evidence of B-dependent antidonor reactivity, who have a median ΔeGFR of −6.8 ml/min per 1.73 m^2^ (IQR 5.7) and mean ΔeGFR of −7.7 ml/min per 1.73 m^2^ (SD ±8.2). Actual *P* = 0.22 by Mann-Whitney *U*. Patients with BFC with no evidence of B-dependent antidonor responses have median ΔeGFR of −0.27 ml/min per 1.73 m^2^ (IQR 13.4) and mean ΔeGFR of −2 ml/min per 1.73 m^2^ (SD ±14.8), compared with those with evidence of B-dependent antidonor reactivity, who have median ΔeGFR of –9.1 ml/min per 1.73 m^2^ (IQR 13.6) and mean ΔeGFR of −9.6 ml/min per 1.73 m^2^ (SD ±13.8). Actual *P* = 0.1 by Mann-Whitney *U*. **Mann-Whitney *U* test.

Figure S5 Changes in eGFR in CAMR subgroup who received protocolized treatment. Box plots showing median with IQR with whiskers showing upper and lower limits of the Modification of Diet in Renal Disease (MDRD) eGFR (**A**) and ΔeGFR (**B**) in the subgroup of patients with CAMR (*n*=15) characterized by having eGFR >20 at time of biopsy, no tubulitis on histological examination of biopsy, and identified as having an ongoing and progressive rise in creatinine, as determined by analysis of reciprocal creatinine plots at the time of first ELISPOT. All were treated with a protocolized treatment regimen, details of which are shown in Table 5. Seven patients stabilized (identified by boxes joined with coarse dotted line). Eight patients failed to stabilize (identified by boxes joined by fine dotted line). Analysis excludes 3 patients who had eGFR <20 at time of biopsy (see Table 5). The differences in the ELISPOT patterns in these 2 subgroups is described in the text. *Points at which values are statistically significant (*P* < 0.05) by Mann-Whitney *U* test.

Figure S6 Experiments to address the role of IL-10 in control of IFN-γ production. Anti–IL-10 monoclonal antibody (to inhibit IL-10) or isotype control was added into the CD8-depleted leukocyte “cone” samples (white, individual 1; gray, individual 2; black, individual 3), 2 showing consistent suppression of IFN-γ production by B cells (white, gray bars) and the third showing B-dependent IFN-γ production (black bars). Frequencies >50/million CD4+ T cells (dotted line on graph) were defined as positive. SFC, spotforming cells. The impact of the antibody after B-cell depletion suggests there is an additional source of IL-10, other than B cells, in the PBMC.

Table S1a Patients excluded from analysis of outcomes because eGFR at first ELISPOT ≤20 ml/min per 1.73 m^2^ OR because follow-up data missing. **Table S1b.** Basic demographics, biopsy results, and eGFR data on the “stable” subgroup with ΔeGFR >median in either PROTCL or BFC group. **Table S1c.** Basic demographics, biopsy results, and eGFR data on the “deteriorating” subgroup with ΔeGFR ≤median in either PROTCL or BFC group.

Table S2a Impact of reducing threshold for positive DSA to >0. **Table S2b.** Lack of association between DSA and outcomes in BFC cohort only. **Table S2c.** Lack of association between DSA and outcomes in PROTCL cohort only.

Table S3 Associations between ELISPOT pattern and outcomes in PROTCL (A) and BFC (B) subgroups.

Table S4 Association between antidonor reactivity based on functional B-cell phenotype and patient outcomes in PROTCL (A) and BFC (B) cohorts.

Table S5 PROTCL cohort – factors used for prediction modeling.

Table S6 BFC cohort – factors used for prediction modeling.

Table S7 Dynamic changes in ELISPOT patterns.

Table S8 Dynamic changes in antiviral antigen ELISPOT patterns and lack of association with outcome.

Table S9 Dynamic changes in antiviral ELISPOT patterns.
