# Supplementary material for: Graft dysfunction in chronic antibody-mediated rejection correlates with B-cell–dependent indirect antidonor alloresponses and autocrine regulation of interferon-γ production by Th1 cells
Source: Kidney Int. 2017 Feb;91(2):477–92. doi: 10.1016/j.kint.2016.10.009 (PMC5258815; doi:10.1016/j.kint.2016.10.009)
Supplement: Supplementary Methods [file mmc1.pdf]

Supplementary file

Methods

Patient recruitment and follow-up

The detailed demographic details of sixty five patients in either protocol biopsy (PROTCL, *n* = 19) or biopsy for cause (BFC, *n* = 46) cohorts were presented in a recent publication (2). Aside from 4 PROTCL-control patients included from the KALIBRE study[1], the others were prospectively enrolled into an observational study at a single centre, the West London Renal and Transplant Centre, London, United Kingdom, between 2005 and 2009. Patients were identified following either a protocol biopsy (PROTCL; *n* = 15) or biopsy for cause (BFC; *n* = 46). In patients who had further biopsies on follow-up, only the first biopsy was used for analysis. Protocol biopsies were performed on all patients transplanted during this period within 6-12 months post-transplantation and at 3 months post-treatment for acute rejection. PROTCL patients had stable graft function, as evaluated by linear regression analysis of serial creatinines (dysfunction defined as negative slope if adjusted $R^2>0.35$ and $p<0.05$) (1) and no significant proteinuria (protein:creatinine ratio < 50 mg/mmol). BFC patients had all been identified as having a rising creatinine or significant proteinuria (≥ 50 mg / mmol) and had a biopsy for diagnostic reasons. BFC patients had all been identified as having a rising creatinine or significant proteinuria (≥ 50 mg / mmol) and had a biopsy for diagnostic reasons showing features of either: positive C4d staining of peritubular capillaries or glomeruli or evidence of chronic Ab-mediated injury. A small control group of BFC patients had graft dysfunction with ≥ 10% interstitial fibrosis / tubular atrophy (IF/TA) on biopsy without features of Ab-mediated injury. All biopsies were reported at the time and then reviewed again for the purposes of this report by one of two experienced renal histopathologists (TC / CR). Patients with acute rejection (>10% rise in

[1] http://www.kcl.ac.uk/lsm/research/divisions/timb/research/tolerance/physicians/biomarker/kalibre.aspx

the two creatinines pre-biopsy and histological appearances of acute cellular or acute Ab-mediated rejection on biopsy) were excluded from the observational study. ABO-incompatible recipients, patients who had had HLA Ab desensitisation treatment pre-transplant, and HBV, HCV and HIV positive patients were excluded. Within the period of the study, all patients eligible for the observational study on the basis of these criteria were asked to give informed consent. Some patients (*n* = 17) were not included in this study despite eligibility because they were enrolled into a randomised controlled trial for the treatment of renal transplant recipients with chronic graft dysfunction and a C4d positive biopsy (RituxiCAN-C4 trial - clinicaltrials.gov identifier NCT00476164): 2 were originally consented to the observational study but then were recruited to the trial and a further 15 patients were directly enrolled into the trial.

Patient management was guided by unit protocols and was not dictated by the observational study. Treatment of the BFC patients was determined clinically. Patients gave consent to use the results of routine clinical investigations, medical notes and laboratory records for research purposes. The Modification of Diet in Renal Disease (MDRD) equation (http://nephron.org/mdrd_gfr_si) was used to calculate the MDRD eGFR from serum creatinines. To minimise the impact of visit to visit variation in eGFR on the estimation of how GFR had changed over time, ΔeGFR was calculated from linear regression analysis of eGFR trends beginning from time of first ELISPOT (time point1) point 1 to end of follow-up, graft failure or 3 years, whichever was the soonest.

Blood was collected from patients within a month of biopsy (time point 1) and 9-12 months later. Data from later samples, collected in a few patients, was included in our first report on this cohort (2), but have not been included here.

Anti-donor antibody testing

Peripheral blood was obtained by standard phlebotomy in plain vacutainers (BD), and allowed to clot. Samples were centrifuged, and serum stored at -80 °C until used. All anti-donor antibody testing was performed at a single laboratory (Hammersmith Hospital), which is a participant in the UK National Quality Assessment Service for Histocompatibility and Immunogenetics, and uses their quality controls to validate the thresholds used for positive and negative antibody testing. Screening for HLA and MICA Ab was performed by flow cytometry using xMAP (Luminex) platform, utilising LABScreen Mixed Bead and single antigen HLA Class I and Class II kits (One Lambda, California, USA) were used to further define specificity as described previously. Cumulative MFI was calculated for all DSA present, when more than one DSA was detected as previously described (3).

Preparation of responder peripheral blood mononuclear cells (PBMC)

Peripheral blood samples were collected by standard phlebotomy (60ml total volume), and processed within 8 hours of venesection. PBMC were isolated by standard density gradient centrifugation using Lymphoprep (Axis-Shield, Stockport, UK). After washing, aliquots were frozen in 10% DMSO with 90% human AB serum (Life Technologies, Paisley, UK), and stored in liquid nitrogen until use. Magnetic bead separation was performed using CD8, CD19 and / or CD25 Dynabeads (Life Technologies); bound cells were discarded, and the negative fraction used in the ELISPOT assay. All viable samples were analysed by ELISPOT.

ELISPOT Assay

Two sets of positive controls were used in every assay. First, to control for antigen processing and presentation, responder cells were stimulated with a mixture of whole recombinant VZV gE (ProSpec-Tany Technogene, Rehovot, Israel), CMV gB and CMV pp65 (both from Abcam, Cambridge, UK) antigens. These three contain the

immunodominant epitopes for both B and T cells from the majority of healthy normals (4-6, 7 and personal communication, J. Breuer). This control was to show that antigen processing and presentation was occurring under the conditions of the assay. The assay therefore tested alloantigen recognition via the indirect pathway. Second, PBMC were also stimulated with anti-CD3/anti-CD28–coated beads (T-Cell Expander, Life Technologies) as a control for cell viability. Negative control wells contained antigen solubilising solution alone (=background). Preparation of donor antigens is described below. Following incubation at 37 °C in 5% CO_2 for 24 hours, plates were washed before addition of alkaline phosphatase-conjugated anti-IFNγ antibody and incubated for 2 h at room temperature. Finally, the plates were washed and developed with substrate solution (BCIP/NBT). The plates were analysed using an automated spot counter and software (AID Elispot Reader system, Germany). All viable samples were analysed. The incremental frequency of spots was calculated by subtracting the negative control wells from the wells containing responder cells with donor antigen. Results are reported as frequency of spot-forming cells / million CD4+ cells; for each sample, T cell percentages were determined by flow cytometry. A threshold of 25 spots / million CD4+ responder cells was chosen as positive after definition by in-house testing and validation in patients and healthy volunteers. [2]
In some assays, a blocking IL-10 monoclonal or isotype control (both from ebioscience, used at 10μg/ml) were added into the assay with CMV gB.

Preparation of Donor antigens

PBMC were rapidly freeze-thawed three times using alternate liquid nitrogen / 37 °C water bath (8). The suspension was checked for lack of integrity of cells, ultracentrifuged at 100,000g for 60 minutes at 4 °C, then resuspended in solubilising solution (6M urea, 2% CHAPS with protease inhibitor (Boehringer Mannheim, Bracknell, UK). Cells from the

[2] Where patients had two or more samples from different time points, these were assessed using the same reagents, under the same conditions and on the same day.

kidney donor were used where available, to provide the full array of HLA and non-HLA antigens. Substitute donors were chosen so they had HLA-A, -B, -C, -DR and –DQ mismatches similar to the actual donor and to reflect the known Ab profile of the recruits, i.e. to include antigens to which patients had DSA but to avoid antigens to which they had non-DSA or avoid antigens from a previous failed transplant. (9).

Analysis of cytokine production

PBMC were incubated under same conditions as for ELISPOT assay with anti-CD46 (generated in house (10)) and anti-CD3 (purified from OKT3 hybridoma), both at 2μg/ml for 22 hours, before assessment by flow cytometry. After washing, cells were stained with titrated amounts of fluorochrome-conjugated monoclonal Ab (CD4-FITC, CD19-APC-Cy7) in PBS with 10% human AB serum for 30 minutes at 4 °C. Ab were obtained from Ebioscience (San Diego, CA), BD Bioscience (Oxford, UK) and Life Technologies. Following staining, the cells were washed twice with PBS and then incubated with Fixable LIVE/DEAD Aqua-fluorescent reactive dye (Life Technologies) for 30 minutes at 4 °C. Cells were washed, fixed for 15 minutes in 1% paraformaldehye, then washed with PBS-5% FCS before acquisition on an LSRII/Fortessa flow cytometer at the BRC Flow Cytometry Laboratory, King's College London with Flowjo software (Treestar Inc). Single living CD4+ cells were gated and analysed for IFNγ and IL-10 positivity using IFNγ (APC) and IL-10 (PE) secretion assay kits (Miltenyi Biotec), according to manufacturer's instructions.

Results & Discussion

Patient groups and outcomes.

Limited ELISPOT and follow-up data were available on 4 PROTCL-control patients from the KALIBRE study, so this report concerns 61 patients[3] from the Hammersmith Hospital. Seven of these patients had an eGFR of <20ml/min/1.73m^2 at time of first ELISPOT so have been excluded from the outcomes analyses. Two others with incomplete follow up data (1 from each of BFC-CAMR/BFC-control) were also excluded (supplementary table 1a), leaving 37 BFC patients and 15 PROTCL patients (total n=52). Relevant features of these patients are in supplementary tables 1b&c.

Since we excluded from our analyses of outcomes all those with an eGFR ≤20 mls/min/1.73m^2, the patients in whom grafts failed during the follow-up period were those with the greatest and most rapid declines in eGFR, a fact confirmed by the demonstration that all 11 graft failure patients appeared in the 'deteriorating' eGFR group (defined as those with a change in eGFR that was greater or equal to the median change within each of the PROTCL and BFC subgroups). By including these patients into the analyses of ΔeGFR, we are able to differentiate between factors associated with significant reductions in eGFR during the period of follow up, irrespective of whether the patients suffer graft failure. Estimated GFRs have been accepted as a clinically relevant endpoint in studies of graft dysfunction in renal transplant recipients (11).

The PROTCL group received no treatment on the basis of their biopsy and was followed for a median (range) of 31.4 (15.5-48.8) months. In this group, the median eGFR at time of first ELISPOT was 49 mls/min/1.73m^2 (Inter quartile range (IQR) 14) with a mean eGFR of 49 (±SD10.8) mls/min/1.73m^2. The median change in eGFR (ΔeGFR) from the time of the biopsy to the end of follow-up was -4.18 mls/min/1.73m^2 (±IQR 8.95) (Figure 1).

BFC patients included in the outcomes analysis were followed for 36 months. The median eGFR in these at time of first ELISPOT was 39.1 mls/min/1.73m^2 (IQR 16.3) with a mean eGFR of 41 (±SD 13.3) ml/min/1.73m^2. Post biopsy, all underwent treatment appropriate to

[3] Hammersmith cohorts: PROTCL-AMR (n=14), PROTCL-control (n=1), BFC-CAMR (n= 38) and BFC-control (n=8)

the diagnosis, which was not dictated by the study. In general, patients in the BFC-CAMR subgroup were treated with optimisation of tacrolimus and MMF (some patients also received rituximab, see below) and those with tubulitis on biopsy received additional corticosteroids. Patients in the BFC-control group with CNI toxicity had a 50% reduction in their dose of calcineurin inhibitor dose. The median ΔeGFR from the time of the first ELISPOT to month 36 in this group was -7.8 mls/min/1.73m^2 (IQR 15) (figure 1). In cases of graft loss, the last recorded creatinine prior to commencing dialysis was used to calculate the MDRD eGFR and ΔeGFR used in this analysis[4].

DSA associations with outcome

There was a significant association, at both time points, between presence of DSA and graft failure (table 1). However, only a minority (41%) of those with a DSA that had a median fluorescence intensity (MFI) >1000 developed graft failure and of those who lost their grafts, only 64% had a DSA >1000, although 90% of those with a negative test (DSA <1000) maintained a functioning graft for the length of the study. Using a lower MFI threshold to define a DSA influenced the strength of the association with graft failure (supplementary table 2a), and it was lost when only the BFC cohort (in whom all the graft failures occurred), or PROTCL cohorts were considered in isolation (supplementary tables 2b&c).

Patients with DSA in which the MFI was >1000 at time point 1 had significantly greater median reductions in eGFR over the course of follow-up (Supplementary figure 2), compared to patients with either no DSA or DSA with MFI <1000 (in whom the Δ*eGFR* appeared no different to patients with no DSA). However, there was considerable overlap, so the presence or absence of DSA did not discriminate patients in the deteriorating or stable subgroups at either time point in either the combined cohort (table 1), nor the BFC

[4] NB: This is different to the previous publication (Shiu et al 2015), in which the ΔeGFRs presented in supplementary tables assumed that eGFR at time of starting dialysis was zero.

or PROTCL groups (supplementary tables 2b&c). We previously reported no association between the presence of DSA and time point 1 ELISPOT patterns (2). This was also true for this smaller cohort and there was no association between presence or absence of DSA and DSR/NDSR on ELISPOT at time point 2, nor between presence or absence of DSA and DSR/NDSR at either time point (data not shown).

Detailed description of changes in ELISPOT in individual patients.

To dissect the basis of observations reported in the main body, and particularly to assess whether functional B cell phenotype was important during these changes, we looked in detail at how the ELISPOT changed in individual patients with two viable samples (supplementary table 7). Strikingly, of nine patients that changed from NDSR to DSR[5], 7/9 involved loss of or change in regulation of IFNγ production and 5/7 of these patieents, (including all three who lost evidence of B cell regulation) were in the deteriorating BFC subgroup. The remaining two patients switched from complete non-responsiveness to either B-dependent DSR without regulation (deteriorating PROTCL subgroup) or developed B-regulated DSR (stable PROTCL subgroup). These data are consistent with the hypothesis that a persistence of 'regulation' despite a change towards detectable anti-donor IFNγ production, can associate with stable graft function.

Ten patients changed from DSR at time point 1 to NDSR at time point 2, and 8/10 were in the stable BFC subgroup. Of these four[6] became completely non-responsive to donor antigens whilst the other four[7] developed or maintained evidence of anti-donor reactivity regulated by B or T cells (or both). Both PROTCL patients also developed evidence of regulation but were in the deteriorating subgroup. These data are consistent with the hypothesis that a change from B-dependent anti-donor reactivity to complete 'non-

[5] Includes patient ID 407 in supplementary table 7
[6] Includes ID 2063 in supplementary table 7.
[7] Includes ID 145 in supplementary table 7

responsiveness' was associated with stability, as opposed to 'gain of regulation' which associated with stability in some but not all patients.

Twelve patients stayed NDSR at both time points. This included four BFC patients who were non-responsive to donor antigens without evidence of regulation at both time points and 2/4 remained stable. Three patients with evidence of regulation at time point 1 became non-responsive at time point 2; two without evidence of B-dependent activity at time point 1 were in the stable subgroup, whereas the third, with evidence of regulated B-dependent anti-donor activity at time point 1, was in the deteriorating subgroup. Two patients shifted from non responsiveness at time point 1 to anti-donor reactivity suppressed by B cells at time point 2 and a third, with evidence of regulated B-dependent anti donor activity at time point 1 lost evidence of B dependence but maintained evidence of regulation at time point 2; all three were in the stable subgroup. The two remaining in this group had evidence of B-dependent reactivity at both time points that was regulated by T cells only, and both were in the deteriorating subgroup.

Finally, 5/6 patients who remained DSR at both time points[8] had a deteriorating eGFR, three of whom had no evidence of any regulation of anti-donor IFNγ production at either time point.

Relating these changes to table 4 in the main manuscript, ten patients who had no evidence of B-cell dependent anti-donor responses at time point 1 and who were either non-responsive or had evidence of active regulation of responses at time point 2 had very little change in their eGFR: 8/10 were in the 'stable' subgroup. Eighteen patients who had evidence of B-dependent anti-donor IFNg production at time point 1, but who developed evidence of regulation or non-responsiveness at time point 2 had intermediate losses in eGFR, and 8/18 were in the 'stable' subgroup. Finally, 9 patients maintained or developed evidence of unregulated B cell-dependent anti-donor IFNg production by time point 2 and

[8] Patient ID 254 showed B-regulated DSR at both time points but was in the BFC deteriorating subgroup.

these had the greatest changes in their eGFR: 7/9 of these were in the deteriorating subgroup.

Importance of IL-10 and regulation of IFNγ in Th-1 CD4+ cells.

Having identified the importance of functional suppression of anti-donor IFNγ production by B cells, it was logical to assess whether IL-10 was involved. In our previous report, we showed that polyclonal B cell stimulation of samples where B cells suppressed IFNγ production in the ELISPOT induced a cytokine response that was skewed towards IL-10 production, compared to samples where B cells presented antigen (2).

Detailed investigation was hampered by a lack of patient samples, but because we had already showed that ELISPOT patterns to a cocktail of viral proteins were similar to those induced by donor antigens (see figure 4, main manuscript), we assessed responses to recombinant CMV protein gpB in PBMC from leukocyte 'cones'. There was a functional B cell phenotype in the CD8-depleted PBMC responses to gpB in 3/5 randomly selected cones (the other 2/5 were non-responsive), two of which showed evidence of a B cell regulated anti-donor IFNγ production, the other showed unregulated B cell-dependent IFNγ production (supplementary figure 6). Addition of an inhibitory anti-IL-10 monoclonal antibody to the CD8-depleted PBMC enhanced the frequency of IFNγ producing spots in the presence of B cells. However, IFNγ production was also significantly enhanced by the anti-IL-10 antibody in B cell depleted samples, suggesting that additional cells, other than B cells, were making IL-10 in these assays.

Autocrine production of IL-10 by Th-1 cells acts as a regulatory mechanism to downregulate IFNγ secretion, as part of the natural life cycle of Th-1 cells (12). The molecular signals involved in the IL-0 autocrine pathway have recently been defined. Intracellular cleavage of complement component C3 by cathepsin L (CTSL) into active C3a and C3b engages their respective receptors C3aR and CD46 to induce IFNg. Thus,

patients with deficiencies in CD46 or C3 suffer from recurrent infections (13, 14). Further signals through CD46 in conjunction with high environmental IL-2 (generated during Th-1 expansion) induce co-expression of IL-10 and begin the (self) regulative phase leading to the termination of the antigen-specific responses. Th-1 cells activated in this way have been shown to interact efficiently with B cells and drive antibody production (15), which may have relevance for our data.

Supplementary References

1. Dudley C, Pohanka E, Riad H*, et al.* Mycophenolate mofetil substitution for cyclosporine a in renal transplant recipients with chronic progressive allograft dysfunction: the "creeping creatinine" study. *Transplantation* 2005; **79:** 466-475.

2. Shiu KY, McLaughlin L, Rebollo-Mesa I*, et al.* B-lymphocytes support and regulate indirect T-cell alloreactivity in individual patients with chronic antibody-mediated rejection. *Kidney Int* 2015; **88:** 560-568.

3. Willicombe M, Brookes P, Sergeant R*, et al.* De novo DQ donor-specific antibodies are associated with a significant risk of antibody-mediated rejection and transplant glomerulopathy. *Transplantation* 2012; **94:** 172-177.

4. Utz U, Britt W, Vugler L*, et al.* Identification of a neutralizing epitope on glycoprotein gp58 of human cytomegalovirus. *Journal of virology* 1989; **63:** 1995-2001.

5. Gonczol E, Plotkin S. Development of a cytomegalovirus vaccine: lessons from recent clinical trials. *Expert opinion on biological therapy* 2001; **1:** 401-412.

6. Smith JG, Liu X, Kaufhold RM*, et al.* Development and validation of a gamma interferon ELISPOT assay for quantitation of cellular immune responses to varicella-zoster virus. *Clinical and diagnostic laboratory immunology* 2001; **8:** 871-879.

7. Arvin AM. Humoral and cellular immunity to varicella-zoster virus: an overview. *The Journal of infectious diseases* 2008; **197 Suppl 2:** S58-60.

8. Baker RJ, Hernandez-Fuentes MP, Brookes PA*, et al.* Loss of direct and maintenance of indirect alloresponses in renal allograft recipients: implications for the pathogenesis of chronic allograft nephropathy. *J Immunol* 2001; **167:** 7199-7206.

9. Sagoo P, Perucha E, Sawitzki B*, et al.* Development of a cross-platform biomarker signature to detect renal transplant tolerance in humans. *J Clin Invest* 2010; **120:** 1848-1861.

10. Wang G, Liszewski MK, Chan AC*, et al.* Membrane cofactor protein (MCP; CD46): isoform-specific tyrosine phosphorylation. *J Immunol* 2000; **164:** 1839-1846.

11. Levey AS, Inker LA, Matsushita K*, et al.* GFR decline as an end point for clinical trials in CKD: a scientific workshop sponsored by the National Kidney Foundation and the US Food and Drug Administration. *Am J Kidney Dis* 2014; **64:** 821-835.

12. Cardone J, Le Friec G, Vantourout P*, et al.* Complement regulator CD46 temporally regulates cytokine production by conventional and unconventional T cells. *Nat Immunol* 2010; **11:** 862-871.

13. Le Friec G, Sheppard D, Whiteman P*, et al.* The CD46-Jagged1 interaction is critical for human TH1 immunity. *Nat Immunol* 2012; **13:** 1213-1221.

14. Liszewski MK, Kolev M, Le Friec G*, et al.* Intracellular complement activation sustains T cell homeostasis and mediates effector differentiation. *Immunity* 2013; **39:** 1143-1157.

15. Fuchs A, Atkinson JP, Fremeaux-Bacchi V*, et al.* CD46-induced human Treg enhance B-cell responses. *Eur J Immunol* 2009; **39:** 3097-3109.
