# Supplementary figures and images for: Graft dysfunction in chronic antibody-mediated rejection correlates with B-cell–dependent indirect antidonor alloresponses and autocrine regulation of interferon-γ production by Th1 cells
Source: Kidney Int. 2017 Feb;91(2):477–92. doi: 10.1016/j.kint.2016.10.009 (PMC5258815; doi:10.1016/j.kint.2016.10.009)

Supplementary Figure 1

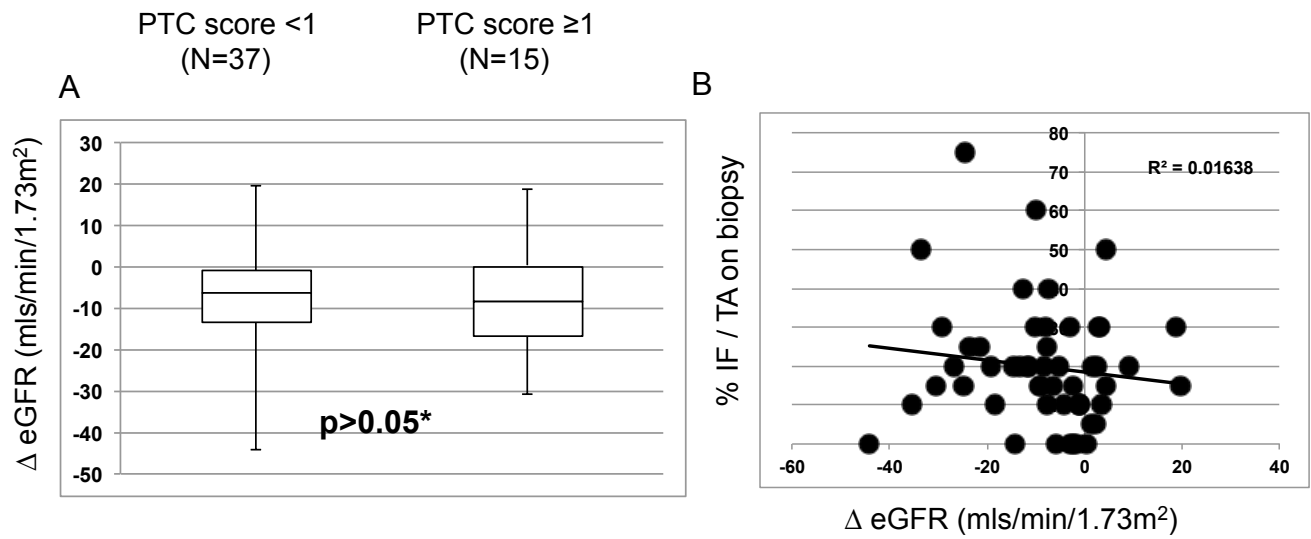

Supplement: Figure S1 — Lack of association between PTC score and ΔeGFR and between IF/TA and ΔeGFR over the course of the study in combined PROTCL and BFC group. (A) Box plots show median with IQR with whiskers showing upper and lower limits of ΔeGFR. Combined group includes all PROTCL and BFC patients, except 9 patients with BFC who either had missing follow-up data (n = 2) or eGFR ≤20 ml/min per 1.73 m2 at time of biopsy (n = 7). Patients with PTC score <1 have median ΔeGFR of −6.85 ml/min per 1.73 m2 (IQR 12.4) and mean ΔeGFR of −8.7 ml/min per 1.73 m2 (SD ±13.1). Patients with PTC score ≥1 have median ΔeGFR of −8.0 ml/min per 1.73 m2 (IQR 16.7) and mean ΔeGFR of −7.2 ml/min per 1.73 m2 (SD ±15.1). *Mann-Whitney U test. (B) Graph shows lack of correlation between IF/TA % on biopsy and ΔeGFR for each of the patients (n = 52) included in this analysis. [file mmc2.pdf]

Supplementary Figure 2

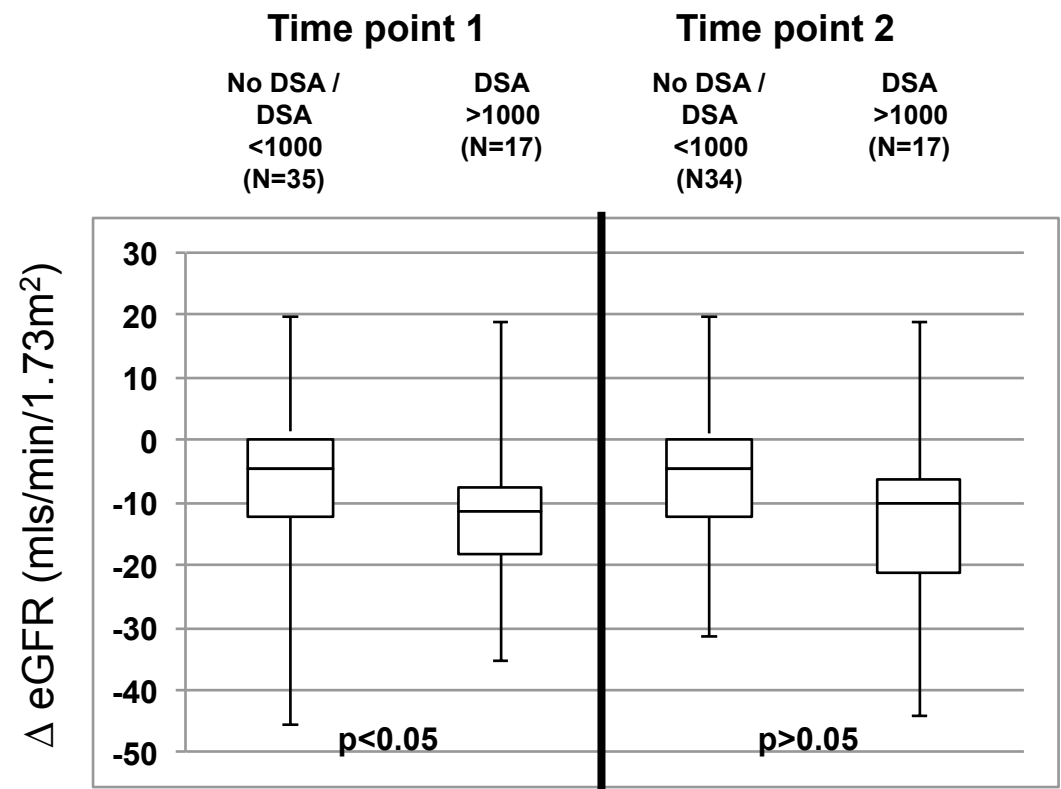

Supplement: Figure S2 — Association between DSA and ΔeGFR over the course of the study in combined cohort. Box plots show median with IQR with whiskers showing upper and lower limits of ΔeGFR. Combined group includes all PROTCL and BFC patients, except 9 patients with BFC who either had missing follow-up data (n = 2), or eGFR ≤20 ml/min 1.73 m2 at time of biopsy (n = 7). Time point 1: Patients with No DSA or DSA with cumulative mean fluorescence intensity of <1000 (n = 35) have median ΔeGFR of −3.19 ml/min per 1.73 m2 (IQR 12.3) and mean ΔeGFR of −6.24 ml/min 1.73 m2 (SD ±13.4). Patients with DSA with cumulative mean fluorescence intensity >1000 (n = 17) have median ΔeGFR of −11.4 ml/min per 1.73 m2 (IQR 10.7) and mean ΔeGFR of −12.4 ml/min per 1.73 m2 (SD ±13.3). Time point 2: Patients with No DSA or DSA with cumulative mean fluorescence intensity of <1000 (n = 34) have median ΔeGFR of −3.69 ml/min per 1.73 m2 (IQR 12.4) and mean ΔeGFR of −5.68 ml/min/1.73 m2 (SD ±11.9). Patients with DSA with cumulative mean fluorescence intensity >1000 have median ΔeGFR of −9.9 ml/min per 1.73 m2 (IQR 15) and mean ΔeGFR of −13.0 ml/min per 1.73 m2 (SD ±16). **Mann-Whitney U test. [file mmc3.pdf]

Supplementary Figure 3

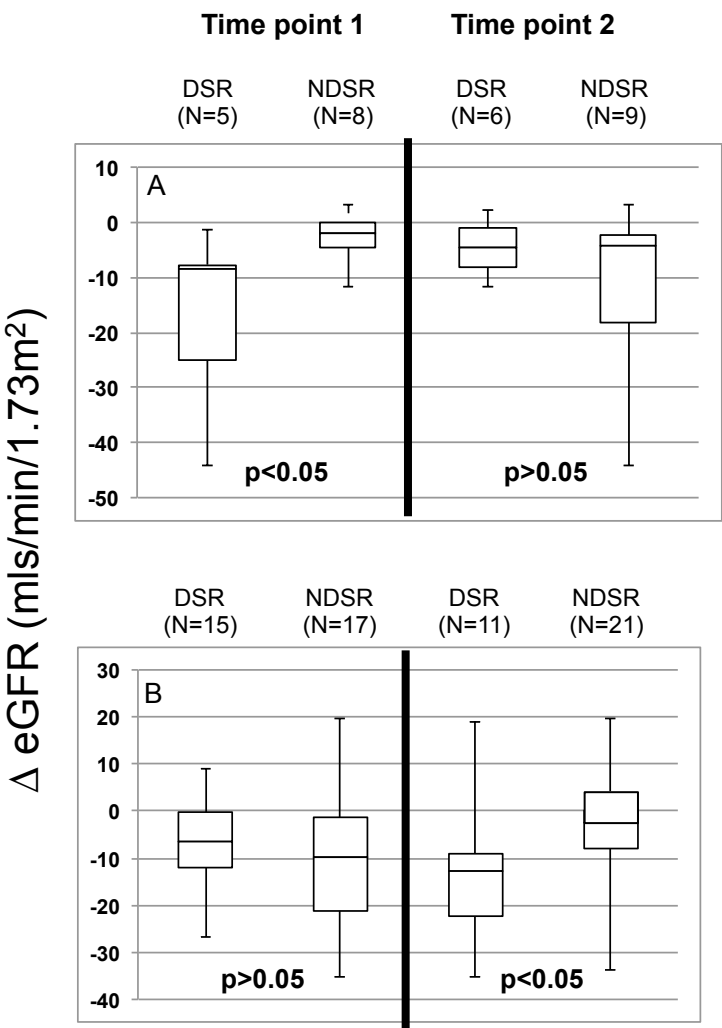

Supplement: Figure S3 — Box plots show median with IQR with whiskers showing upper and lower limits of ΔeGFR in PROTCL (A) and BFC (B) subgroups. Time point 1: PROTCL patients with DSR have median ΔeGFR of −8.34 ml/min per 1.73 m2 (IQR 17.4) and mean ΔeGFR of −17.3 ml/min per 1.73 m2 (SD ±17.4), compared with those with NDSR who have a median ΔeGFR of −2.03 ml/min per 1.73 m2 (IQR 6.2) and mean ΔeGFR of −2.38 ml/min per 1.73 m2 (SD ±4.9). Patients with BFC with DSR have median ΔeGFR of −6.4 ml/min per 1.73 m2 (IQR 11.7) and mean ΔeGFR of −7.1 ml/min per 1.73 m2 (SD ±10.7), compared with those with NDSR who have median ΔeGFR of –9.9 ml/min per 1.73 m2 (IQR 20.1) and mean ΔeGFR of −10.3 ml/min per 1.73 m2 (SD ±17.1). Actual P = 0.46. Time point 2: PROTCL patients with DSR have median ΔeGFR of −4.5 ml/min per 1.73 m2 (IQR 7.2) and mean ΔeGFR of −4.6 ml/min per 1.73 m2 (SD ±5.4), compared to those with NDSR who have median ΔeGFR of −4.2 ml/min per 1.73 m2 (IQR 15.8) and mean ΔeGFR of −10.9 ml/min per 1.73 m2 (SD ±15.5). Patients with BFC with DSR have median ΔeGFR of −12.7ml/min per 1.73 m2 (IQR 13.5) and mean ΔeGFR of −13.5 ml/min per 1.73 m2 (SD ±14.6), compared with those with NDSR who have median ΔeGFR of –2.5 ml/min per 1.73 m2 (IQR 12.3) and mean ΔeGFR of −2.4 ml/min per 1.73 m2 (SD ±12.1). Actual P = 0.01. **Mann-Whitney U test. [file mmc4.pdf]

Supplementary Figure 4

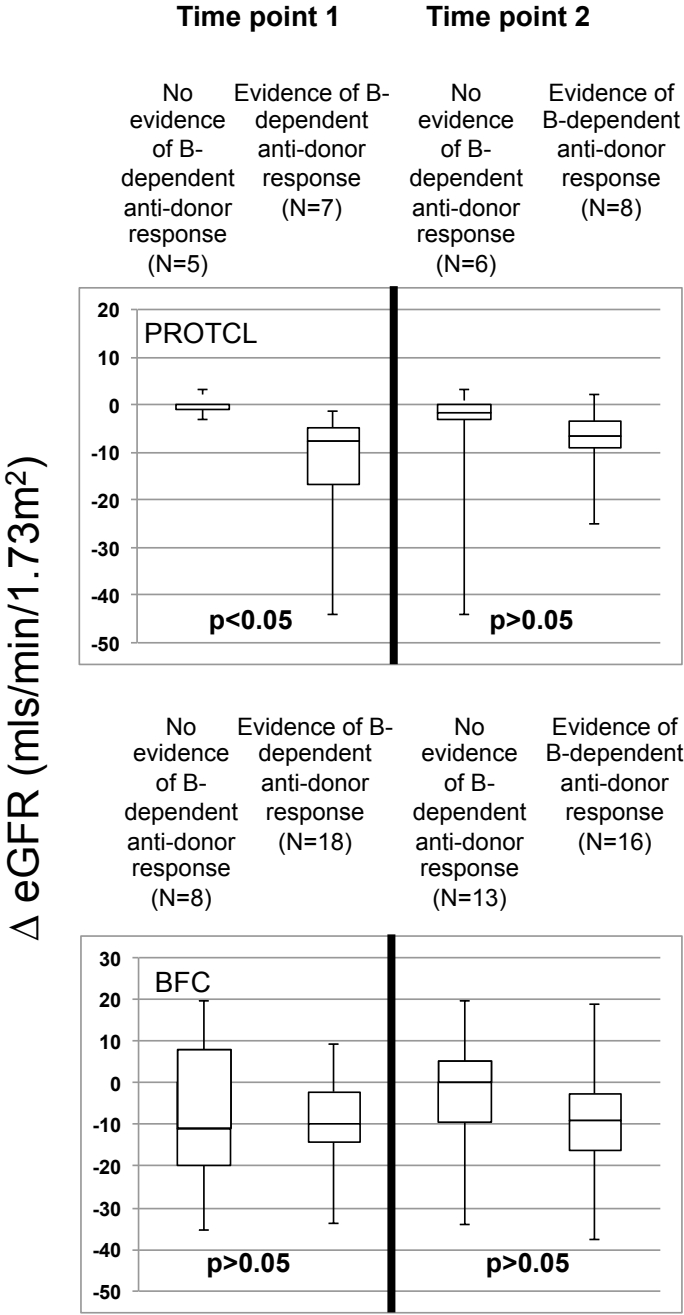

Supplement: Figure S4 — Box plots show median with IQR with whiskers showing upper and lower limits of ΔeGFR in PROTCL (upper panel) and BFC (lower panel) subgroups. Time point 1: PROTCL patients with no evidence of B-dependent antidonor responses have median ΔeGFR of +1.5 ml/min per 1.73 m2 (IQR 3) and mean ΔeGFR of −0.54 ml/min per 1.73 m2 (SD ±2.6), compared with those with evidence of B-dependent antidonor reactivity, who have a median ΔeGFR of −7.7 ml/min per 1.73 m2 (IQR 11.7) and mean ΔeGFR of −13.8 ml/min per 1.73 m2 (SD ±15.4). Patients with BFC with no evidence of B-dependent antidonor responses have median ΔeGFR of −11.1 ml/min per 1.73 m2 (IQR 27.6) and mean ΔeGFR of −6.9 ml/min per 1.73 m2 (SD ±19.7), compared with those with evidence of B-dependent antidonor reactivity, who have median ΔeGFR of –9.7 ml/min per 1.73 m2 (IQR 12) and mean ΔeGFR of −10.3 ml/min per 1.73 m2 (SD ±12.4). Actual P = 0.88 by Mann-Whitney U. Time point 2: PROTCL patients with no evidence of B-dependent antidonor responses have median ΔeGFR of −1.7 ml/min per 1.73 m2 (IQR 3.9) and mean ΔeGFR of −7.7 ml/min per 1.73 m2 (SD ±18), compared with those with evidence of B-dependent antidonor reactivity, who have a median ΔeGFR of −6.8 ml/min per 1.73 m2 (IQR 5.7) and mean ΔeGFR of −7.7 ml/min per 1.73 m2 (SD ±8.2). Actual P = 0.22 by Mann-Whitney U. Patients with BFC with no evidence of B-dependent antidonor responses have median ΔeGFR of −0.27 ml/min per 1.73 m2 (IQR 13.4) and mean ΔeGFR of −2 ml/min per 1.73 m2 (SD ±14.8), compared with those with evidence of B-dependent antidonor reactivity, who have median ΔeGFR of –9.1 ml/min per 1.73 m2 (IQR 13.6) and mean ΔeGFR of −9.6 ml/min per 1.73 m2 (SD ±13.8). Actual P = 0.1 by Mann-Whitney U. **Mann-Whitney U test. [file mmc5.pdf]

Supplementary Figure 5

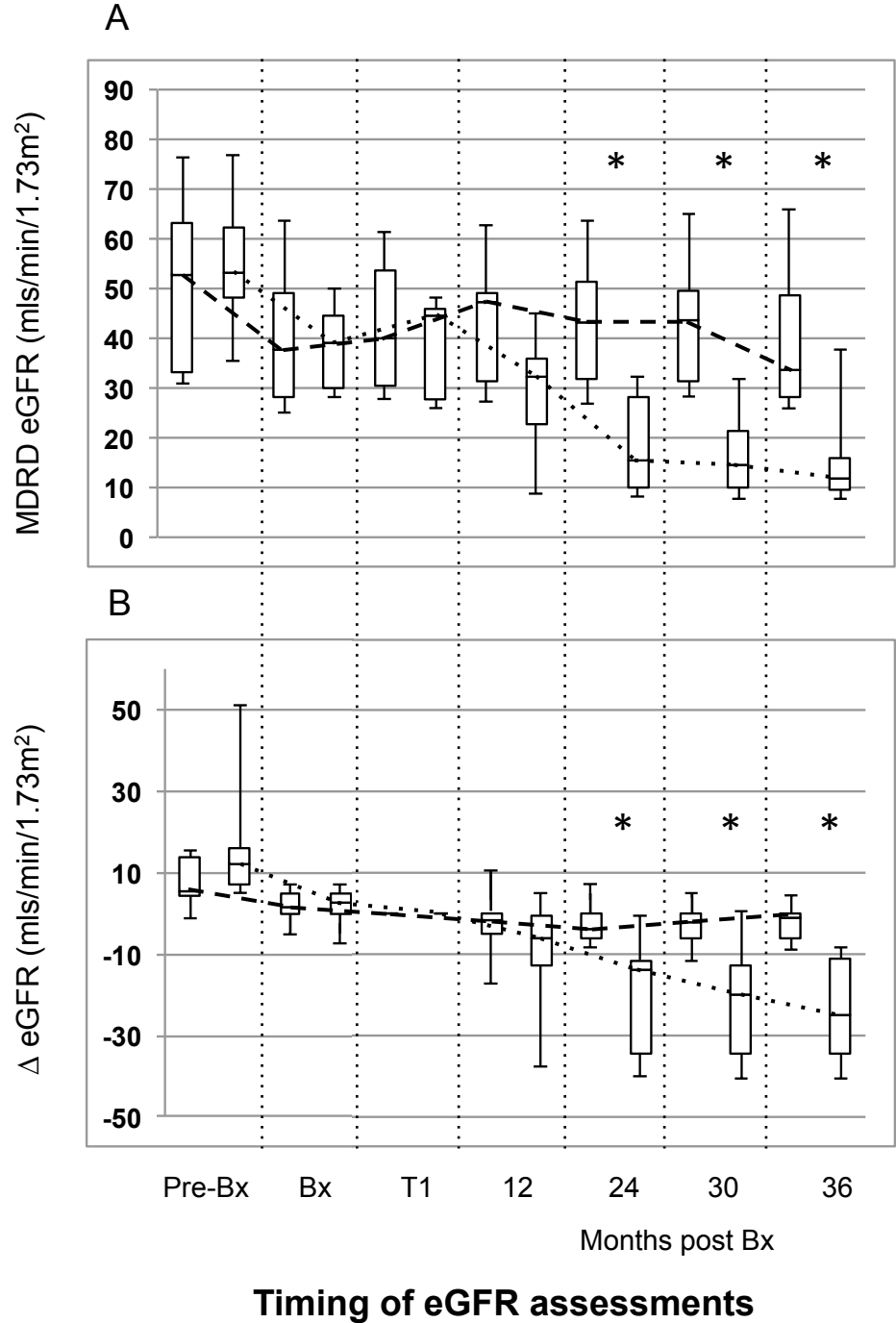

Supplement: Figure S5 — Changes in eGFR in CAMR subgroup who received protocolized treatment. Box plots showing median with IQR with whiskers showing upper and lower limits of the Modification of Diet in Renal Disease (MDRD) eGFR (A) and ΔeGFR (B) in the subgroup of patients with CAMR (n=15) characterized by having eGFR >20 at time of biopsy, no tubulitis on histological examination of biopsy, and identified as having an ongoing and progressive rise in creatinine, as determined by analysis of reciprocal creatinine plots at the time of first ELISPOT. All were treated with a protocolized treatment regimen, details of which are shown in Table 5. Seven patients stabilized (identified by boxes joined with coarse dotted line). Eight patients failed to stabilize (identified by boxes joined by fine dotted line). Analysis excludes 3 patients who had eGFR <20 at time of biopsy (see Table 5). The differences in the ELISPOT patterns in these 2 subgroups is described in the text. *Points at which values are statistically significant (P < 0.05) by Mann-Whitney U test. [file mmc6.pdf]

Supplementary figure 6

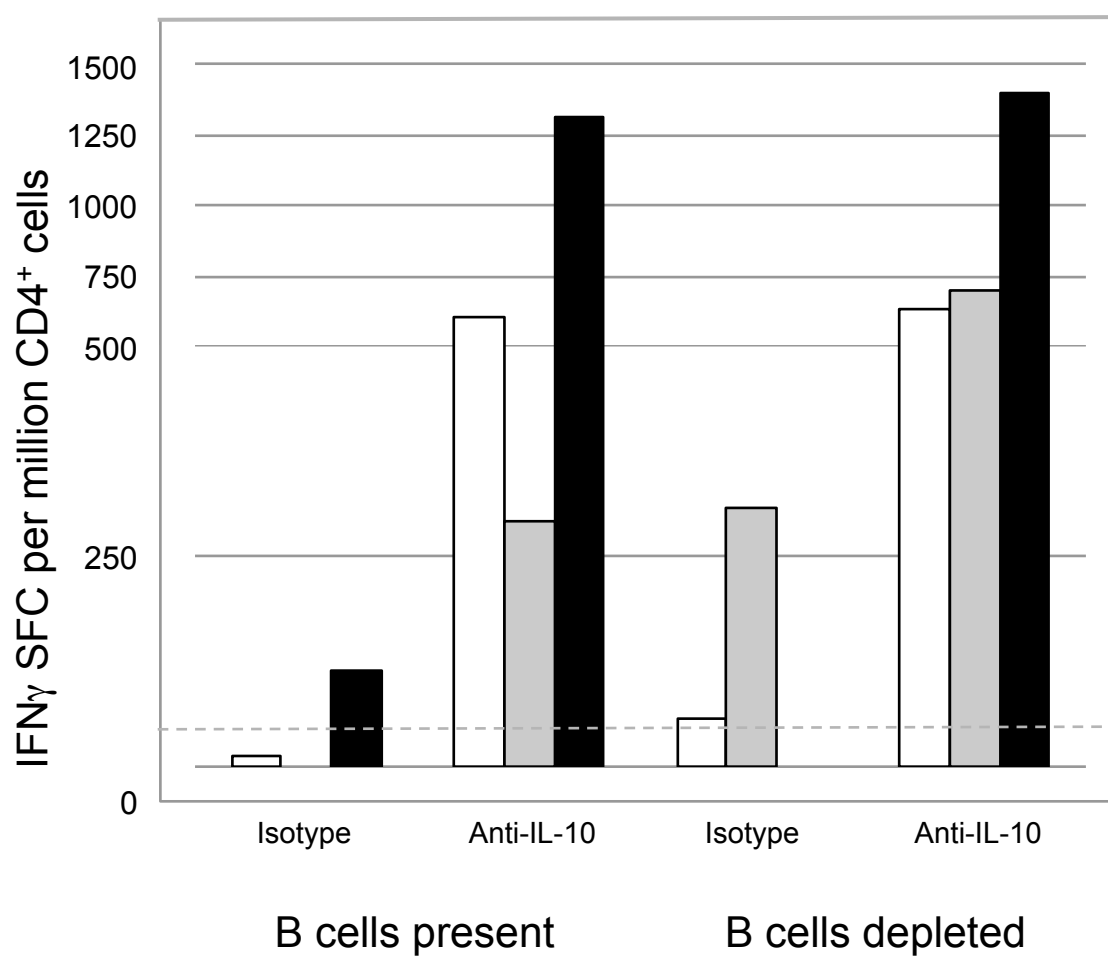

Supplement: Figure S6 — Experiments to address the role of IL-10 in control of IFN-γ production. Anti–IL-10 monoclonal antibody (to inhibit IL-10) or isotype control was added into the CD8-depleted leukocyte “cone” samples (white, individual 1; gray, individual 2; black, individual 3), 2 showing consistent suppression of IFN-γ production by B cells (white, gray bars) and the third showing B-dependent IFN-γ production (black bars). Frequencies >50/million CD4+ T cells (dotted line on graph) were defined as positive. SFC, spotforming cells. The impact of the antibody after B-cell depletion suggests there is an additional source of IL-10, other than B cells, in the PBMC. [file mmc7.pdf]
