# Supplementary material for: Graft dysfunction in chronic antibody-mediated rejection correlates with B-cell–dependent indirect antidonor alloresponses and autocrine regulation of interferon-γ production by Th1 cells
Source: Kidney Int. 2017 Feb;91(2):477–92. doi: 10.1016/j.kint.2016.10.009 (PMC5258815; doi:10.1016/j.kint.2016.10.009)
Supplement: Table S1a — Patients excluded from analysis of outcomes because eGFR at first ELISPOT ≤20 ml/min per 1.73 m2 OR because follow-up data missing. Table S1b. Basic demographics, biopsy results, and eGFR data on the “stable” subgroup with ΔeGFR >median in either PROTCL or BFC group. Table S1c. Basic demographics, biopsy results, and eGFR data on the “deteriorating” subgroup with ΔeGFR ≤median in either PROTCL or BFC group. [file mmc8.pdf]

Supplementary tables

Supplementary Table 1a.

Patients excluded from analysis of outcomes because eGFR at first ELISPOT ≤20 mls/min/1.73m^2 OR because follow-up data missing

ID	Age (yr)	Sex	Biopsy Subgroup	MDRD eGFR at First ELISPOT	PCR>50	Cumulative DSA MFI		C4d (ptc) score	C4d (g) score	aTG (g≥1)	PTCitis (ptc≥1)	cg ≥ 1 / cv ≥ 1	IF / TA (%)	Δ MDRD eGFR
						Time point 1	Time point 2							
807	50.4	F	BFC-CAMR Graft failure	15.2	Y	0	0	1	1	N	Y	Y / Y †	50	-9.4
2006	51.7	F	BFC-CAMR Graft failure	19.6	Y	0	2132	1	2	Y	N	Y / Y	15	-9.2
2010	41.1	F	BFC-CAMR Graft failure	15.1	Y	7849	4738	3	3	Y	Y	N / Y †	50	-4.5
787	56.8	M	BFC-CAMR	56	-*	2642	*	1	0	N	Y	N / Y	30	*
1339	48.3	F	BFC-CAMR Graft failure	18.4	Y	279	88	0	0	Y	N	Y / N	0	-5.6
1132	58.2	F	BFC-ACR Graft Failure	11.4	Y	6378	0	2	3	Y	N	Y / Y	50	-1.7
278	44.8	F	BFC-Control	26.5	Y	0	*	0	0	N	N	N / Y	25	*
832	39.1	M	BFC-Control Graft failure	19.6	Y	0	393	0	0	N	N	N / N	40	-3.5
1448	59.4	M	BFC-CAMR Graft Failure	18.3	Y	350	**	2	1	N	N	Y / Y $	20	-1.40

* no data

† Majority of PTC have >5 layers of basement membrane on EM.

Supplementary Table 1b.

Basic demographics, biopsy results and eGFR data on the ‘stable’ subgroup with ΔeGFR >median in either PROTCL or BFC group

ID	Age (yr)	Sex	Biopsy Subgroup	MDRD eGFR at First ELISPOT	PCR >50	Cumulative DSA MFI		C4d (ptc) score	C4d (g) score	aTG (g≥1)	PTC itis (ptc≥1)	cg ≥ 1/cv ≥ 1	IF / TA (%)	Δ MDRD eGFR
						Time point 1	Time point 2							
739	71.3	F	BFC-CAMR	28.4	N	0	4634	0	0	N	N	Y / Y*	50	4.28
165	53.5	M	BFC-CAMR	27.8	N	0	0	1	2	N	Y	Y / Y	20	2.67
326	44.1	M	BFC-CAMR	32.1	Y	4263	4803	0	0	Y	Y	Y / Y	30	-3.02
145	46.0	M	BFC-Control	70	N	0	0	0	2	N	N	N / Y	20	9.10
397	48.1	M	BFC-CAMR	61.3	Y	0	382	0	1	Y	N	Y / N $	10	3.61
438	51.4	M	BFC-CAMR	46.1	Y	3809	2579	0	1	Y	N	Y / Y *	15	-6.43
841	50.6	M	BFC-CAMR	40	N	5149	11896	1	0	N	N	Y / N	15	4.24
965	42.4	M	BFC-CAMR	61.1	N	0	0	1	0	N	N	Y / N	0	0.27
1030	52.8	M	BFC-Control	48.1	N	0	300	0	0	N	N	N / N	5	1.34
1275	41.9	F	BFC-Control	29.2	N	0	0	0	0	N	N	N / Y	15	-2.50
2063	40.4	F	BFC-Control	35.7	N	0	0	0	0	N	N	N / **	0	-1.86
158	28.8	M	BFC-Control	54.9	N	0	0	0	0	N	N	N / N	20	-5.26
654	32.0	M	BFC-CAMR	38.3	N	0	9434	0	0	N	Y	N / Y ®	30	2.84
664	29.6	F	BFC-CAMR	43.3	Y	2411	164	2	3	Y	Y	N / N	30	18.82
676	40.2	M	BFC-CAMR	32	Y	15700	6604	0	0	N	Y	N / Y ®	25	-7.78
2009	68.8	M	BFC-CAMR	82.7	Y	0	0	0	2	N	Y	Y / N	10	-1.32
2019	67.4	M	BFC-CAMR	70.7	Y	129	0	1	3	N	N	Y / N	15	19.68
736	50.0	F	BFC-CAMR +tubulitis	30.8	Y	16239	31063	3	2	N	N	Y / Y $	40	-7.56
170	61.2	M	PROTCL-AMR	33	N	0	413	2	0	N	N	N / N	10	-1.24
407	64.2	F	PROTCL-AMR	44	N	0	0	0	0	Y	N	N / N	10	-0.90

1450	57.6	F	PROTCL-AMR	42	N	0	0	0	2	Y	N	N / N	30	3.20
1451	55.7	M	PROTCL-AMR	37	N	0	0	2	2	N	N	N / N	20	1.49
2001	49.6	F	PROTCL-AMR	49	N	0	0	1	3	Y	Y	N / N	5	2.10
2003	46.7	F	PROTCL-AMR	49	N	0	0	0	2	N	N	N / N	<10	-2.42
2005	30.1	F	PROTCL-AMR	60	N	0	0	2	2	N	N	N / N	<5	-3.19
			Median	43.3		-	-	-	-	-	-	-	15	0.26
			IQR	21.9		-	-	-	-	-	-	-	10	5.7
			Number (%) positive ***	--	8 (32)	6(24)	7 (29)	10 (40)	13 (52)	7 (28)	7(28)	10 (40) / 9 (38)	-	-
			Number (%) negative	-	17 (68)	19 (76)	17 (71)	15 (60)	12 (48)	18 (72)	18 (72)	15 (60) / 15 (62)	-	-
			Median score (in positive group)	-		-	-	2	2	-	-	-	-	-

* Features of chronic TMA with glomerulopathy (+/- arterial lesions).

Some biopsies showed PTCBMML (>5 layers) by EM: $ some PTCBMML present. † Majority of PTC have >5 layers of BM. ® Repeat biopsies on 2 patients during follow-up; C4d (PTC) >2, C4d (g) 1, PTC 1, g1, cg >1, cv2, IF/TA >30%,EM $

** no data

*** for DSA, number (%) > with MFI 1000

Supplementary Table 1c.

Basic demographics, biopsy results and eGFR data on the 'deteriorating' subgroup with ΔeGFR ≤median in either PROTCL or BFC group

ID	Age (yr)	Sex	Biopsy Subgroup	MDRD eGFR at First ELISPOT	PCR>50	Cumulative DSA MFI		C4d (ptc) score	C4d (g) score	aTG (g≥1)	PTCitis (ptc≥1)	cg ≥ 1 / cv ≥ 1	IF / TA (%)	Δ MDRD eGFR
						Time point 1	Time point 2							
399	52.1	F	BFC-CAMR	45.7	N	2800	0	0	1	Y	N	Y / Y	20	-14.68
635	61.0	M	BFC-CAMR<br>Graft failure	45.3	Y	3800	5851	2	2	Y	N	Y / N *	10	-35.34
254	41.3	M	BFC-CAMR<br>Graft failure	32	Y	0	0	1	2	N	Y	N / Y	40	-12.66
392	54.2	M	BFC-CAMR<br>Graft failure	48.2	Y	3128	2540	1	2	N	N	Y / Y $	50	-33.74
1364	62.3	F	BFC-CAMR	28	Y	0	0	0	0	Y	N	Y / Y	20	-19.13
1404	64.3	M	BFC-CAMR<br>Graft failure	44.1	Y	0	0	0	0	Y	Y	N / N	15	-30.36
2002	37.8	M	BFC-CAMR<br>Graft failure	26	Y	811	0	0	0	N	N	Y / Y $	75	-24.60
2062	45.3	M	BFC-CAMR<br>Graft failure	39.1	Y	4150	14892	2	3	Y	Y	Y / Y	25	-21.38
223	37.8	M	BFC-CAMR<br>+tubulitis<br>Graft failure	47.2	Y	2867	3897	3	3	Y	Y	Y / N	0	-14.41
516	42.2	M	BFC-CAMR<br>+tubulitis<br>Graft failure	20.6	N	3087	1123	2	3	Y	Y	N / N $	30	-8.01
1423	37.8	F	BFC-CAMR<br>Graft failure	22.4	Y	2323	4997	2	3	N	Y	Y / Y	60	-9.93
864	45.5	M	BFC-CAMR<br>+tubulitis	35.2	Y	1752	4875	2	3	Y	N	N / Y $	20	-11.41
835	35.7	M	BFC-CAMR<br>Graft failure	46.4	Y	3158	1953	1	2	N	Y	N / N	30	-29.10
1440	28.5	M	BFC-CAMR	62.6	Y	0	0	0	2	Y	N	N / N	15	-9.4
459	39.9	F	BFC-Control	31.1	Y	0	0	0	0	N	N	N / Y	30	-10.10
861	51.9	F	BFC-CAMR	27.1	Y	0	111	2	0	Y	N	Y / Y*	25	-23.60
31	49.5	F	BFC-CAMR<br>Graft failure	39.3	Y	0	500	0	1	Y	N	Y / Y $	20	-26.75
1438	52.0	M	BFC-CAMR	31.1	Y	208	707	0	0	Y	N	N / N †	15	-9.12
2037	65.5	M	BFC-CAMR	51.5	Y	3589	**	1	2	N	N	N / Y †	20	-13.29
61	48.2	M	PROTCL-AMR	56	N	0	0	2	2	Y	N	N / N	10	-4.18
497	42.0	F	PROTCL-AMR	47	N	98	816	0	0	Y	N	N / N	10	-7.70

958	57.0	F	PROTCL-AMR	58	N	0	1302	0	0	Y	N	N / N	0	-44.15
1187	49.0	M	PROTCL-AMR	70	N	0	0	1	2	N	N	N / N	<5	-5.82
1442	36.0	M	PROTCL-AMR	48	N	167	144	3	3	N	N	N / N	20	-11.70
2030	56.4	F	PROTCL-AMR	32	N	0	0	2	0	N	N	N / N	20	-8.34
1997	50.0	M	PROTCL-AMR	61	N	3575	4465	3	3	N	Y	N / N	10	-18.26
1444	60.1	F	PROTCL-Control	50	N	0	0	0	0	N	N	N / N	15	-25.02
			Median	45.3		-	-	-	-	-	-	-	20	-14.4
			IQR	17.6		-	-	-	-	-	-	-	15	15.2
			Number (%) positive***	-	17 (63)	12 (44)	11 (42)	16 (59)	17 (63)	14 (52)	8 (30)	10 (37) / 13 (48)	-	-
			Number (%) negative	-	10 (37)	15 (56)	15 (58)	11 (41)	10 (37)	13 (48)	19 (70)	17 (63) / 14 (52)	-	-
			Median score (in positive group)	-		-	-	2	2	-	-	-	-	-

* Features of chronic TMA with glomerulopathy (+/- arterial lesions).

Some biopsies showed PTCBMML (>5 layers) by EM: $ some PTCBMML present. † Majority of PTC have >5 layers of BM. ® Repeat biopsies on 2 patients during follow-up; C4d (PTC) >2, C4d (g) 1, PTC 1, g1, cg >1, cv2, IF/TA >30%,EM $

** no data

*** for DSA, number (%) > with MFI 1000
