# Supplementary material for: Graft dysfunction in chronic antibody-mediated rejection correlates with B-cell–dependent indirect antidonor alloresponses and autocrine regulation of interferon-γ production by Th1 cells
Source: Kidney Int. 2017 Feb;91(2):477–92. doi: 10.1016/j.kint.2016.10.009 (PMC5258815; doi:10.1016/j.kint.2016.10.009)
Supplement: Table S2a — Impact of reducing threshold for positive DSA to >0. Table S2b. Lack of association between DSA and outcomes in BFC cohort only. Table S2c. Lack of association between DSA and outcomes in PROTCL cohort only. [file mmc9.pdf]

Supplementary table 2a - impact of reducing threshold for positive DSA to >0

Entire cohort (n=52)	MFI of DSA	Number of samples		P value *	Number of samples		P value *
		Graft failure	No graft failure		Deteriorating eGFR (≤median)	Stable eGFR (>median)	
**Time point 1**	>0 (n=22)	8	14	**<u>0.04</u>**	15	7	0.05
	0 (n=30)	3	27		12	18	
**Time point 2**	>0 (n=26)	8	18	0.17	15	11	0.41
	0 (n=25)	3	22		11	14	
**Overall****	>0 (n=30)	9	21	0.09	18	12	0.26
	0 (n=22)	2	20		9	13	

Supplementary table 2b – Lack of association between DSA and outcomes in BFC cohort only

BFC cohort (n=37)	MFI of DSA	Number of samples		P value *	Number of samples		P value *
		Graft failure	No graft failure		Deteriorating eGFR (≤median)	Stable eGFR (>median)	
**Time point 1**	>1000 (n=16)	7	9	0.15	10	6	0.32
	0 or <1000 (n=21)	4	17		9	12	
**Time point 2**	>1000 (n=15)	7	8	0.14	8	7	1
	0 or <1000 (n=21)	4	17		10	11	
**Overall****	>1000 (n=18)	7	11	0.3	10	8	0.75
	0 or <1000 (n=19)	4	15		9	10	

Supplementary table 2c – Lack of association between DSA and outcomes in PRTCOL cohort only

PROTCL cohort (n=15)	MFI of DSA	Number of samples		P value *	Number of samples		P value *
		Graft failure	No graft failure		Deteriorating eGFR (≤median)	Stable eGFR (>median)	
**Time point 1**	>1000 (n=1)	0	1	1	1	0	1
	0 or <1000 (n=14)	0	14		7	7	
**Time point 2**	>1000 (n=2)	0	2	1	2	0	0.5
	0 or <1000 (n=13)	0	13		6	7	
**Overall****	>1000 (n=2)	0	2	1	2	0	0.5
	0 or <1000 (n=13)	0	13		6	7	

* Fisher exact test
**Overall i.e. DSA at either time point 1 or 2 or both

Graft Failure:
Sensitivity of DSA>0: Time point 1 8/11=73%,
Positive predictive value of DSA>0: time point 1 8/23=35%.
Negative predictive value of DSA 0: Time point 1 26/29=90%.
Specificity: Time point 1 26/41=63%.
