# Supplementary material for: Graft dysfunction in chronic antibody-mediated rejection correlates with B-cell–dependent indirect antidonor alloresponses and autocrine regulation of interferon-γ production by Th1 cells
Source: Kidney Int. 2017 Feb;91(2):477–92. doi: 10.1016/j.kint.2016.10.009 (PMC5258815; doi:10.1016/j.kint.2016.10.009)
Supplement: Table S3 — Associations between ELISPOT pattern and outcomes in PROTCL (A) and BFC (B) subgroups. [file mmc10.pdf]

Supplementary table 3 – Associations between ELISPOT pattern and outcomes in PROTCL (A) and BFC (B) subgroups.

A	ELISPOT pattern	Number of samples		P value *	Number of samples		P value *
		Graft failure	No Graft failure		Deteriorating eGFR (≤median)	Stable eGFR (>median)	
**Time point 1**	DSR (n=5)	0	5	1	4	1	0.26
	NDSR (n=8)	0	8		3	5	
**Time point 2**	DSR (n=6)	0	6	1	3	3	1
	NDSR (n=9)	0	9		5	4	
**Change to or maintenance of:** **	DSR (n=6)	0	6	1	3	3	1
	NDSR (n=7)	0	7		4	3	

B	ELISPOT pattern	Number of samples		P value *	Number of samples		P value *
		Graft failure	No Graft failure		Deteriorating eGFR (≤median)	Stable eGFR (>median)	
**Time point 1**	DSR (n=15)	3	12	0.26	7	8	0.74
	NDSR (n=17)	7	10		9	8	
**Time point 2**	DSR (n=11)	5	6	0.09	8	3	**0.03**
	NDSR (n=21)	3	17		6	15	
**Change to or maintenance of:** **	DSR (n=9)	5	4	**0.02**	7	2	**0.01**
	NDSR (n=18)	2	16		4	14	

* Fisher exact test
** in paired samples only (i.e samples in which ELISPOTS available at both time points)
