# Supplementary material for: Graft dysfunction in chronic antibody-mediated rejection correlates with B-cell–dependent indirect antidonor alloresponses and autocrine regulation of interferon-γ production by Th1 cells
Source: Kidney Int. 2017 Feb;91(2):477–92. doi: 10.1016/j.kint.2016.10.009 (PMC5258815; doi:10.1016/j.kint.2016.10.009)
Supplement: Table S4 — Association between antidonor reactivity based on functional B-cell phenotype and patient outcomes in PROTCL (A) and BFC (B) cohorts. [file mmc11.pdf]

**Supplementary table 4: Association between anti-donor reactivity based on functional B cell phenotype and patient outcomes in PROTCL (A) and BFC (B) cohorts**

**A**

| | ELISPOT pattern | Number of samples | | P value * | Number of samples | | P value * |
|---|---|---|---|---|---|---|---|
| | | Graft failure | No Graft failure | | Deteriorating eGFR (≤median) | Stable eGFR (>median) | |
| **Time point 1** | No evidence of B-dependence (n=5) | 0 | 5 | 1 | 0 | 5 | **0.02** |
| | Evidence of B-dependence (n=7) | 0 | 7 | | 6 | 1 | |
| **Time point 2** | No evidence of B-dependence (n=6) | 0 | 6 | 1 | 1 | 5 | 0.1 |
| | Evidence of B-dependence (n=8) | 0 | 8 | | 6 | 2 | |
| **Change to or maintenance of:**** | No evidence of B-dependence (n=5) | 0 | 5 | 1 | 1 | 4 | 0.09 |
| | Evidence of B-dependence (n=8) | 0 | 8 | | 6 | 2 | |

**B**

| | ELISPOT pattern | Number of samples | | P value * | Number of samples | | P value * |
|---|---|---|---|---|---|---|---|
| | | Graft failure | No Graft failure | | Deteriorating eGFR (≤median) | Stable eGFR (>median) | |
| **Time point 1** | No evidence of B-dependence (n=8) | 2 | 7 | 0.7 | 4 | 4 | 1 |
| | Evidence of B-dependence (n=18) | 6 | 11 | | 10 | 8 | |
| **Time point 2** | No evidence of B-dependence (n=13) | 1 | 12 | 0.09 | 4 | 9 | 0.26 |
| | Evidence of B-dependence (n=16) | 6 | 10 | | 9 | 7 | |
| **Change to or maintenance of:**** | No evidence of B-dependence (n=11) | 1 | 10 | 0.17 | 3 | 8 | 0.1 |
| | Evidence of B-dependence (n=13) | 5 | 8 | | 8 | 5 | |

* Fisher exact test
