# Supplementary material for: Graft dysfunction in chronic antibody-mediated rejection correlates with B-cell–dependent indirect antidonor alloresponses and autocrine regulation of interferon-γ production by Th1 cells
Source: Kidney Int. 2017 Feb;91(2):477–92. doi: 10.1016/j.kint.2016.10.009 (PMC5258815; doi:10.1016/j.kint.2016.10.009)
Supplement: Table S5 — PROTCL cohort – factors used for prediction modeling. [file mmc12.pdf]

Supplementary Table 5: PROTCL cohort – factors used for prediction modelling

Model	Factors included	AUC	AUC 95% CI
Demographic	Age<br>Sex<br>Ethnicity (Caucasian/ Non-Caucasian)<br>Previous acute rejection<br>MDRD at time of protocol biopsy	0.741	0.46 – 1.0
Recipient Factors from time of Transplant	Age at transplant<br>Type of transplant<br>Time on dialysis pre-transplant<br>Time on transplant waiting list pre-transplant	0.833	0.59 – 1.0
Donor Factors	Class I mismatches<br>Class II mismatches<br>Cold ischaemic time<br>Donor age<br>Donor sex<br>Donor Cause of death	0.796	0.56 – 1.0
HLA	HLA status (at time of protocol biopsy)<br>MICA status (-"-)<br>HLA MFI<br>DSA MFI	0.833	0.63 – 1.0
Protocol Biopsy	C4d positivity in glomeruli<br>C4d positivity in peritubular capillaries<br>Microvascular inflammation (g or ptc ≥1)<br>Interstitial fibrosis / tubular atrophy	0.843	0.63 – 1.0
ELISPOT	DSR<br>B-dependent reactivity post-CD25 depletion<br>Evidence of regulation by B cells	0.912	0.76 – 1.0
