# Supplementary material for: Graft dysfunction in chronic antibody-mediated rejection correlates with B-cell–dependent indirect antidonor alloresponses and autocrine regulation of interferon-γ production by Th1 cells
Source: Kidney Int. 2017 Feb;91(2):477–92. doi: 10.1016/j.kint.2016.10.009 (PMC5258815; doi:10.1016/j.kint.2016.10.009)
Supplement: Table S6 — BFC cohort – factors used for prediction modeling. [file mmc13.pdf]

Supplementary Table 6: BFC cohort – factors used for prediction modelling

Model	Factors included	AUC	AUC 95% CI
Demographic	Age<br>Sex<br>Ethnicity (Caucasian/ Non-Caucasian)<br>Previous acute rejection<br>MDRD at time of biopsy for cause	0.757	0.58 – 0.93
Other Factors	Number of transplant<br>Type of transplant<br>PRA pre-transplant (categorised as >70 or <70)<br>Proteinuria at time of biopsy<br>Deteriorating 1/Cr at time of biopsy<br>Protocolised treatment with Tac/MMF/Rituximab<br>Class I HLA MM, Class II HLA MM, donor age, donor sex, donor cause of death, delayed graft function?	0.817	0.63 – 0.96
HLA Ab	HLA status<br>MICA status<br>HLA MFI at time of biopsy<br>DSA MFI at time of biopsy<br>MICA weak / strong at time of biopsy	0.668	0.49 – 0.84
Biopsy for Cause	C4d positivity in glomeruli<br>C4d positivity in peritubular capillaries<br>Microvascular inflammation (g or ptc ≥1)<br>Transplant glomerulopathy (cg i.e. double contours)<br>Interstitial fibrosis / tubular atrophy	0.804	0.66 – 0.94
ELISPOT	B-dependent DSR<br>B-dependent reactivity post-CD25 depletion	0.527	0.53 – 0.91
