# Supplementary material for: Graft dysfunction in chronic antibody-mediated rejection correlates with B-cell–dependent indirect antidonor alloresponses and autocrine regulation of interferon-γ production by Th1 cells
Source: Kidney Int. 2017 Feb;91(2):477–92. doi: 10.1016/j.kint.2016.10.009 (PMC5258815; doi:10.1016/j.kint.2016.10.009)
Supplement: Table S7 — Dynamic changes in ELISPOT patterns. [file mmc14.pdf]

Supplementary Table 7: Dynamic changes in ELISPOT patterns.

				Time Point 2									
				NDSR						DSR			T2 ND or Not fully interpre table
				No regulation	'Breg'		'Treg', 'Breg'		'Treg'	Bdep			
			Interpretation based on B cell phenotype	No regulation	'Breg': only when CD25+ cells present	'Breg': when CD25+ cells present / absent	'Breg': only when CD25+ cells absent	'Breg' when CD25 present / 'Bdep' when CD25 absent	'Bdep': only when CD25+ cells absent	'Bdep': when CD25 present, 'Breg' when CD25+ cells absent	'Bdep' when CD25+ cells present / absent	'Bdep': only when CD25+ cells present	
Time point 1	NDSR	No regul ation	No regulation	1364<br>399<br>739<br>2019	407 (P)***	1451 (P)	1450 (P)					1442 (P)†	736
		'Breg'	'Breg': only when CD25+ cells present	2005 (P)						2001 (P)	835†		
			'Breg': when CD25+ cells present and absent	664								2062	
		Treg', 'Breg'	'Breg': only when CD25+ cells absent								635		
			'Breg' when CD25 present BUT 'Bdep' when CD25 absent,	**2063			165	**254***<br>**145	61 (P)				1404
		'Treg' 'Bdep'	'Bdep' only when CD25+ cells absent	392					1187 (P)	223<br>676		2009	
	DSR	'Bdep'	'Bdep' when CD25 present, 'Breg' when CD25+ cells absent										31*
			'Bdep' when CD25+ cells present and absent		958 (P)			326	1444 (P)<br>438	864		2030 (P)<br>459	2002<br>2037<br>1440
			'Bdep': only when CD25+ cells present	158†<br>841†<br>1030†					397			497 (P)<br>170 (P)	
	T1 ND or Not fully interpretable			965	1438	2003 (P)			516		861	654	1275#<br>1423#<br>1997 (P)

** DSR Breg at time point 1

*** DSR with Breg at time point 2
† these samples had incomplete testing at T1, making it difficult to ascribe a particular pattern, and are therefore included in the not done/not viable row in table 2a, but difference between T1 &T2 nevertheless discernible on T2 sample alone.
# these samples were NDSR T1
Patients highlighted in yellow are in the ‘stable’ subgroup, whereas those highlighted in red are in the ‘deteriorating’ subgroup.
