# Supplementary material for: Graft dysfunction in chronic antibody-mediated rejection correlates with B-cell–dependent indirect antidonor alloresponses and autocrine regulation of interferon-γ production by Th1 cells
Source: Kidney Int. 2017 Feb;91(2):477–92. doi: 10.1016/j.kint.2016.10.009 (PMC5258815; doi:10.1016/j.kint.2016.10.009)
Supplement: Table S8 — Dynamic changes in antiviral antigen ELISPOT patterns and lack of association with outcome. [file mmc15.pdf]

Supplementary table 8: Dynamic changes in anti-viral antigen ELISPOT patterns and lack of association with outcome.

<table>
<tr><td colspan="3" rowspan="3">Interpretation based on B cell phenotype</td><td colspan="4">Time Point 2**</td></tr>
<tr><td></td><td colspan="2">Evidence of Regulation</td><td>No evidence of regulation</td></tr>
<tr><td>No response</td><td>Regulated anti-viral response without evidence of B-dependency</td><td>B dependent anti-viral response with evidence of regulation</td><td>Unregulated B-dependent anti-viral response</td></tr>
<tr><td rowspan="4">Time point 1*</td><td rowspan="2">No evidence of B-dependent anti-viral response</td><td>No response</td><td colspan="3" rowspan="2">8 patients: 3 stable, 5 deteriorating</td><td rowspan="4">7 patients: 3 stable, 4 deteriorating***</td></tr>
<tr><td>Regulated anti-viral response without evidence of B-dependency</td></tr>
<tr><td rowspan="2">Evidence of B-dependent anti-viral response</td><td>B dependent anti-vral response with evidence of regulation</td><td colspan="3" rowspan="2">15 patients: 9 stable, 6 deteriorating</td></tr>
<tr><td>Unregulated B-dependent anti-viral response</td></tr>
</table>

* 10 additional patients with time point 2 samples had time point 1 samples that were not either not done or not fully interpretable, so they are not included in this analysis
** 5 additional patients with time point 1 samples had time point 2 samples that were not either not done or not fully interpretable, so they are not included in this analysis
4 patients had neither time point 1 or 2 samples that could be interpreted by B cell phenotype so they are not included here.
2 patients had ELISPOT patterns that had a B-independent phenotype at one or other time points,
***Comparison of stable and deteriorating patients in each group: p=0.63 Fisher Exact Probability 3x2 test

Refer to supplementary table 9 for full details of all results.
