# Supplementary material for: Graft dysfunction in chronic antibody-mediated rejection correlates with B-cell–dependent indirect antidonor alloresponses and autocrine regulation of interferon-γ production by Th1 cells
Source: Kidney Int. 2017 Feb;91(2):477–92. doi: 10.1016/j.kint.2016.10.009 (PMC5258815; doi:10.1016/j.kint.2016.10.009)
Supplement: Table S9 — Dynamic changes in antiviral ELISPOT patterns. [file mmc16.pdf]

Supplementary table 9: Dynamic changes in anti-viral ELISPOT patterns.

		Time Point 2										
	Interpretation based on B cell phenotype	No regulation	'Breg': only when CD25+ cells present	'Breg': when CD25+ cells present / absent	'Breg': only when CD25+ cells absent (Treg)	'Breg' when CD25 present / 'Bdep' when CD25 absent (Treg)	'Bdep': only when CD25+ cells absent (Treg)	'Bdep': when CD25 present, 'Breg' when CD25+ cells absent	'Bdep' when CD25+ cells present / absent	'Bdep': only when CD25+ cells present	No B cell phenotype	ND or Not interpretable
Time point 1	No regulation	392		1364§		407(P)**<br>1442(P)**			739			
	'Breg': only when CD25+ cells present	2005(P)<br>497(P)						676		2062	2001(P)†§	
	'Breg': when CD25+ cells present and absent			958 (P)								2002
	'Breg': only when CD25+ cells absent (Treg)											
	'Breg' when CD25 present BUT 'Bdep' when CD25 absent (Treg)					*864**†<br>*145**<br>*2063	*1444(P)**†	*1451(P)†<br>*165†	*397†<br>*635	*459		1404
	'Bdep' only when CD25+ cells absent (Treg)							2019§				
	'Bdep' when CD25 present, 'Breg' when CD25+ cells absent	170(P)				61 (P)•¶						31†
	'Bdep' when CD25+ cells present and absent			438**¶§		326**†¶		223†				2009†
	'Bdep': only when CD25+ cells present		2030 (P)†			664**†		399†	254	1450 (P)†		2037
	No B cell phenotype						*1187(P)¶					
	ND or Not interpretable	1438		965§<br>861<br>158**		2003(P)**<br>516**			835†§<br>1997 (P)†<br>654<br>1030			736<br>841<br>1275<br>1423

Unless indicated otherwise, results with patterns in the shaded areas are NDSR, those in unshaded areas, DSR.
T1 Bindep when CD25 present
* DSR T1: ** DSR T2: ¶ evidence of Treg T1: § evidence of Treg T2
† actual patterns in these samples: 1444 T2, 1997 T2, 223 T1, 326 T1, 397 T2, 835 T2, 2009 T1 'Bindep when CD25present, Bdep when CD25 absent' : 1450 T1 and T2, 2030 T1, 664 T1 'Bdep when CD25 present, Bindep when absent': 1451 T2, 31 T1, 399 T2 'Bindep when CD25present, Breg when CD25 absent': 2001 T2 'Bindep when CD25present, NR when CD25 absent': 165 T1, 397 T1, 664 T2, 864 T1 and T2 'Breg when CD25present, Bindep when absent'.
Patients highlighted in yellow are in the 'stable' subgroup, whereas those highlighted in red are in the 'deteriorating' subgroup.
